# Giant Small‐Molecule Donors With Controlled Backbone Planarity Afford High‐Performance and Photostable Organic Solar Cells

**DOI:** 10.1002/advs.202512427

**Published:** 2025-11-19

**Authors:** Hyerin Jeon, Seunghoon Song, Jin‐Woo Lee, Yun‐Hi Kim, Bumjoon J. Kim

**Affiliations:** ^1^ Department of Chemical and Biomolecular Engineering Korea Advanced Institute of Science and Technology (KAIST) Daejeon 34141 Republic of Korea; ^2^ Department of Chemistry and RIMA Gyeongsang National University Jinju 528258 Republic of Korea

**Keywords:** all small‐molecule organic solar cells, giant small‐molecule donor, glass‐transition temperature, organic solar cells, photo‐stability

## Abstract

High power conversion efficiency (PCE) and long‐term stability are critical requirements for the commercialization of organic solar cells (OSCs). Small‐molecule donors (SMDs) are promising due to easy purification and excellent batch‐to‐batch reproducibility, but they suffer from poor morphological stability associated with their fast diffusion kinetics. Here, new giant small‐molecule donors (GSMDs) are designed that combine increased molecular sizes with excellent optoelectronic properties, enabling both efficient and photostable OSCs. To optimize their thermal and electrical properties, we tune the backbone planarity of GSMDs by controlling the orientation of alkyl side chains. Specifically, two GSMDs with different molecular configurations are synthesized: 1) **GSMD‐*syn***, where alkyl side chains are aligned in the same direction, and 2) **GSMD‐*anti***, with oppositely oriented side chains. The anti‐configuration reduces steric hindrance between backbone units, thereby enhancing crystallinity and charge transport. Consequently, **GSMD‐*anti***:Y6‐based OSCs achieve a high PCE of 15.4%, significantly outperforming those based on conventional SMD (**BTR‐Cl**:Y6, PCE = 13.4%) and **GSMD‐*syn***:Y6 (PCE = 11.9%). Furthermore, ternary devices incorporating **GSMD‐*anti*** reach a PCE of 16.5%, which, to the best of our knowledge, is among the highest reported values for GSMD‐based OSCs. Importantly, **GSMD‐*anti***:Y6 OSCs exhibit significantly enhanced photostability (*t*_80%_ = 1510 h), compared to **BTR‐Cl**:Y6 OSCs (*t*_80%_ = 60 h), attributed to suppressed molecular diffusion resulting from the larger molecular size of **GSMD‐*anti***.

## Graphical abstract

Efficient and photostable organic solar cells (OSCs) are developed using giant small‐molecule donors (GSMDs) with controlled backbone planarity. By tuning alkyl side chain orientation, anti‐configured GSMD improved crystallinity and charge transport, yielding PCE of 15.4% and superior stability (t_80%_ > 1500 h). A ternary all‐small‐molecule blend further increased the PCE to 16.5%, highlighting the potential of GSMD‐based OSCs.

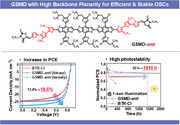

## 1 Introduction

Organic solar cells (OSCs) have garnered significant interest as a promising technology for next‐generation renewable energy sources due to their unique attributes, such as lightweight, semi‐transparency, and solution processability.^[^ ^1^, ^2^, ^3^, ^4^ ^]^ Over the past few decades, OSCs have achieved remarkable progress with power conversion efficiencies (PCEs) exceeding 19–20%, primarily driven by the advancement of polymer donor (*P*_D_) and small‐molecule acceptor (SMA) systems.^[^ ^5^, ^6^, ^7^, ^8^ ^]^ However, the inherent batch‐to‐batch variability of *P*_D_s poses a potential obstacle to their scalability and industrial viability.^[^ ^9^, ^10^, ^11^, ^12^ ^]^

Small‐molecule donors (SMDs) offer a compelling alternative to *P*_D_s, with key advantages such as well‐defined molecular structures, ease of purification, and excellent batch‐to‐batch consistency.^[^ ^13^, ^14^, ^15^, ^16^, ^17^ ^]^ Moreover, their strong self‐assembly behavior can promote well‐ordered intermolecular assembly in the photoactive layer, leading to improved charge transport and reduced voltage losses in OSCs.^[^ ^18^, ^19^, ^20^, ^21^ ^]^ These advantages have led to growing interest in the development of all‐small‐molecule organic solar cells (SM‐OSCs), which incorporate both SMDs and SMAs.^[^ ^22^, ^23^ ^]^ Although SM‐OSCs have demonstrated promising efficiencies (> 16%) and processing advantages, their limited long‐term operational stability remains a critical barrier to commercialization.^[^ ^24^, ^25^, ^26^ ^]^ This instability primarily stems from the degradation of the optimized blend morphology, caused by the rapid diffusion of SMD molecules under external stresses such as light and heat.^[^ ^27^, ^28^ ^]^ Due to their small molecular size, SMDs typically exhibit high diffusion coefficient (*D*) and low glass transition temperatures (*T*_g_s), both of which contribute to increased molecular mobility and a stronger tendency for phase separation over time, ultimately reducing device stability.^[^ ^29^, ^30^, ^31^ ^]^

Giant small‐molecule donors (GSMDs), with their increased molecular sizes, offer the potential to combine the advantages of both *P*_D_s and SMDs for the development of efficient and stable OSCs.^[^ ^32^, ^33^, ^34^, ^35^, ^36^, ^37^, ^38^, ^39^ ^]^ Their extended conjugation lengths may lead to reduced diffusion kinetics and higher *T*_g_ compared to conventional SMDs, resulting in improved device stability under thermal‐ and photo‐stress.^[^ ^40^, ^41^, ^42^, ^43^ ^]^ At the same time, their well‐defined molecular structures ensure superior batch‐to‐batch reproducibility compared to *P*_D_s.^[^ ^44^, ^45^, ^46^, ^47^, ^48^ ^]^ For instance, Wei group recently reported the development of two GSMDs (G‐Dimer‐D1 and G‐Dimer‐D2) with different linker structures, synthesized through the dimerization of SMD molecules (MPhS‐C6).^[^ ^44^ ^]^ They showed that OSCs based on the GSMD with stronger intermolecular assembly (G‐Dimer‐D2) exhibited significantly enhanced long‐term stability while maintaining a high PCE of 15.7%, outperforming their SMD‐based counterparts. However, the PCEs of GSMD‐based OSCs still fall short of those achieved by state‐of‐the‐art *P*_D_:SMA‐based OSCs, thus requiring further improvement. Furthermore, it is imperative to develop design principles to guide the development of advanced GSMD materials.

One of the primary challenges in designing GSMDs is preserving the high optoelectronic properties of their SMD backbones, as the enlarged molecular dimensions and increased steric hindrance in GSMDs often compromise crystallinity and charge mobility. To overcome this limitation, enhancing backbone planarity is essential to maintain the robust intermolecular packing and well‐connected fibril networks necessary for efficient charge transport.^[^ ^49^, ^50^, ^51^, ^52^ ^]^ A critical structural parameter that significantly influences molecular conformation and planarity is the positioning and orientation of the alkyl side chains.^[^ ^53^, ^54^, ^55^, ^56^ ^]^ The spatial arrangement of these side chains affects steric interactions between building blocks, thereby determining the overall planarity of the conjugated backbone. For example, Yan group reported that a polymer acceptor with regularly oriented side chains (PYF‐T‐*o*) exhibited significantly higher crystallinity and charge mobility compared to its counterpart with randomly oriented side chains (PYF‐T), resulting in an improved PCE in OSCs (15.2 vs 14.0%).^[^ ^53^ ^]^ In addition, Ge group demonstrated that an SMD featuring alkyl side chains arranged in opposite directions (BT‐2F) had reduced steric hindrance and enhanced backbone planarity, leading to superior molecular packing and charge transport compared to BTEC‐2F, in which the side chains were aligned in the same direction.^[^ ^54^ ^]^ Therefore, we expect that controlling the positioning and orientation of alkyl side chains in GSMDs could serve as an effective handle to optimize backbone planarity and achieve superior optoelectronic performance.

In this study, we design a new series of GSMDs (**GSMD‐*anti*** and **GSMD‐*syn***) with controlled molecular conformations and backbone planarity and develop efficient and photostable SM‐OSCs (**GSMD‐*anti***:Y6, *t*_80%_ lifetime > 1500 h). Importantly, we demonstrate the critical role of backbone planarity in GSMD design through a direct structural comparison between two side‐chain configurations: 1) **GSMD‐*syn***, where the alkyl side chains are aligned in the same direction, and 2) **GSMD‐*anti***, where side chains are aligned in the opposite direction. Our results show that the opposite alkyl side chain arrangement in **GSMD‐*anti*** effectively reduces steric hindrance between backbone units, which in turn enhances crystallinity and charge transport in photoactive films. Consequently, OSCs based on **GSMD‐*anti***:Y6 achieve a high PCE of 15.4%, significantly outperforming those based on the conventional SMD (**BTR‐Cl**:Y6, PCE = 13.4%) and **GSMD‐*syn***:Y6 (PCE = 11.9%). Importantly, the **GSMD‐*anti***:Y6 devices exhibit markedly enhanced photostability (*t*_80%_ lifetime = 1510 h) compared to **BTR‐Cl**:Y6 (*t*_80%_ lifetime = 60 h), which we attribute to reduced molecular diffusion arising from the increased molecular size of **GSMD‐*anti***. Then, we further improve the PCE value of the **GSMD‐*anti***‐based OSCs to 16.5% by constructing an all‐small‐molecule ternary blend while maintaining good photostability (*t*_80%_ lifetime = 1470 h). To the best of our knowledge, this represents one of the highest reported PCEs for GSMD‐based OSCs. Thus, this study provides important insights into the design of GSMDs in achieving OSCs with both high PCE and long‐term stability.

## 2 Results and Discussion

### 2.1 Basic Material Characteristics

**Figure 1:**
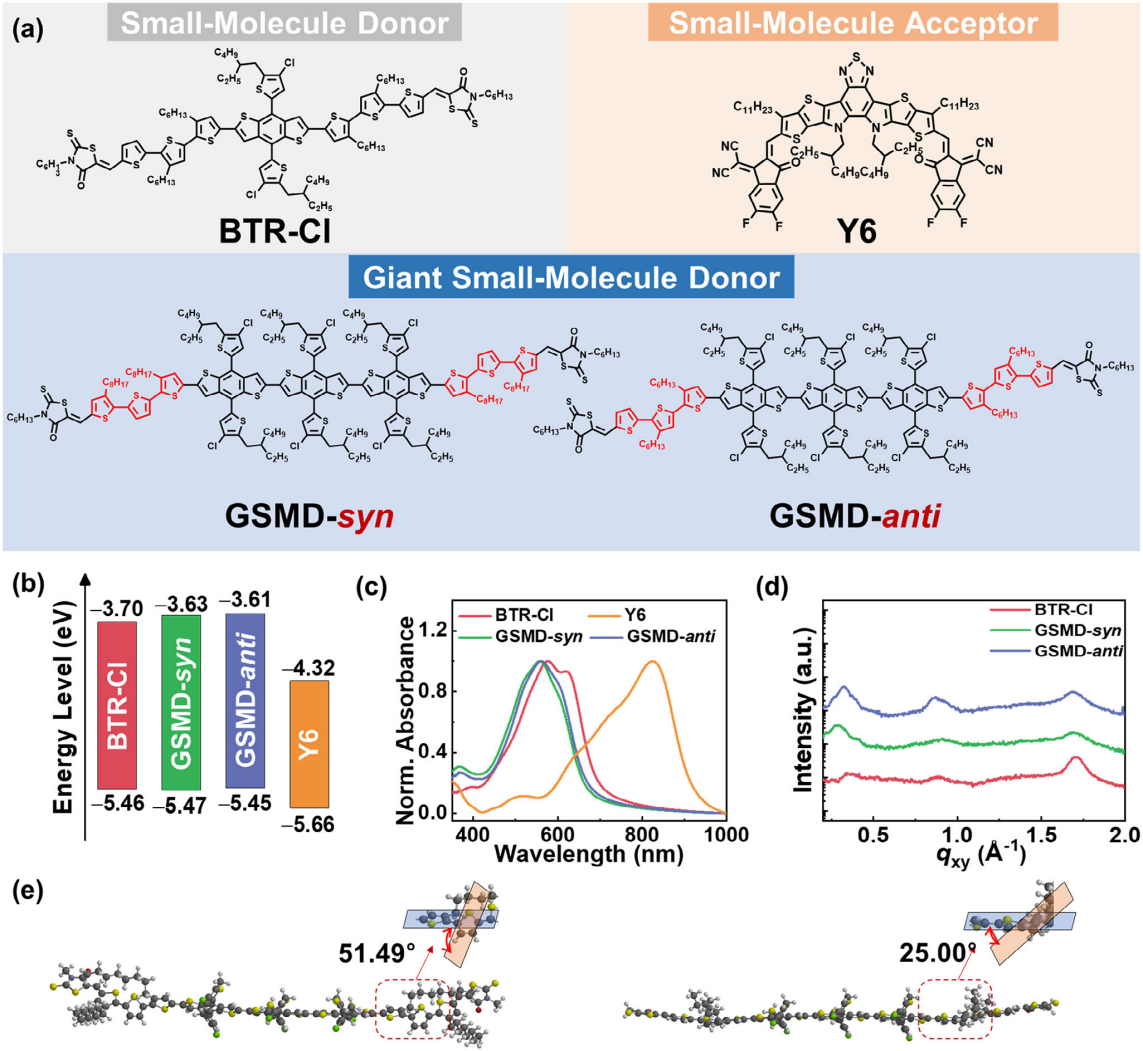
a) Molecular structures of donors and acceptors used in this study. b) Energy level alignment and c) UV–vis absorption spectra in the film of donors and acceptors. d) GIXS line‐cut profiles in the in‐plane direction of the donors. e) Optimized molecular conformations of the GSMDs obtained from DFT simulations.

The chemical structures of the photoactive materials used in this study are shown in **Figure** **1**a. (5*Z*,5*Z*″)‐5,5′‐(((4,8‐Bis(4‐chloro‐5‐(2‐ethylhexyl)thiophen‐2‐yl)benzo[1,2‐*b*:4,5‐*b*″]dithiophene‐2,6‐diyl)bis(3′,3′′‐dihexyl‐[2,2′:5′,2′″‐terthiophene]‐5′″,5‐diyl))bis(methanylylidyne))bis(3‐hexyl‐2‐thioxothiazolidin‐4‐one) (**BTR‐Cl**) is a well‐known high‐performance SMD consisting of benzo[1,2‐*b*:4,5‐*b*″]dithiophene (BDT) core unit, terthiophene spacer units, and rhodanine‐based terminal dye units.^[^ ^18^ ^]^ **BTR‐Cl** offers several advantages, including 1) excellent charge transport enabled by its high crystallinity, 2) effective complementary absorption with SMAs, and 3) easy purification and superior batch‐to‐batch reproducibility. However, due to its relatively small molecular size, **BTR‐Cl** suffers from fast diffusion kinetics, raising concerns about the morphological and long‐term stabilities of OSCs.

To address this limitation, we designed a new series of GSMDs ((5*Z*,5*Z′*)‐5,5′‐(((4,4′,4″,8,8′,8″‐hexakis(4‐chloro‐5‐(2‐ethylhexyl)thiophen‐2‐yl)‐[2,2′:6′,2″‐terbenzo[1,2‐*b*:4,5‐*b′*]dithiophene]‐6,6″‐diyl)bis(3,3″‐dihexyl‐[2,2′:5′,2″‐terthiophene]‐5″,5‐diyl))bis(methanylylidene))bis(3‐hexyl‐2‐thioxothiazolidin‐4‐one) (**GSMD**‐***anti***) and (5*Z*,5*Z*′)‐5,5′‐(((4,4′,4″,8,8′,8″‐hexakis(4‐chloro‐5‐(2‐ethylhexyl)thiophen‐2‐yl)‐[2,2′:6′,2″‐terbenzo[1,2‐*b*:4,5‐*b′*]dithiophene]‐6,6″‐diyl)bis(3,3″‐dioctyl‐[2,2′:5′,2″‐terthiophene]‐5″,5‐diyl))bis(methanylylidene))bis(3‐hexyl‐2‐thioxothiazolidin‐4‐one) (**GSMD‐*syn***)) by expanding the number of BDT core units from one to three. We anticipated that increasing the molecular size of the GSMDs would raise the *T*_g_ and lower the *D*, thereby significantly enhancing the morphological stability of the blend films. In particular, to optimize the impact of side‐chain orientation on the crystallinity and optoelectronic properties of GSMDs, we designed and synthesized two GSMD variants with distinct side‐chain configurations: one with side chains oriented in opposite directions (**GSMD**‐***anti***) and the other with side chains aligned in the same direction (**GSMD‐*syn***) (Schemes S1 and S2, Supporting Information). The detailed synthetic procedures are provided in the Supporting Information. The successful synthesis of the materials was confirmed using nuclear magnetic resonance (NMR) and matrix‐assisted laser desorption/ionization time of flight (MALDI‐ToF) analyses (Figures S1–S12, Supporting Information). Thermogravimetric analysis (TGA) revealed that both GSMDs had high decomposition temperatures (*T*_d_s) of 402 and 404 °C, respectively, indicating the good material stability against heating compared to **BTR‐Cl** (Figure S13, Supporting Information). We used 2,2′‐((2*Z*,2′*Z*)‐((12,13‐bis(2‐ethylhexyl)‐3,9‐diundecyl‐12,13‐dihydro‐[1,2,5]thiadiazolo[3,4‐*e*]thieno[2″,3″:4′,5′]thieno[2′,3′:4,5]pyrrolo[3,2‐*g*]thieno[2′,3′:4,5]thieno[3,2‐*b*]indole‐2,10‐diyl)bis(methanylylidene))bis(5,6‐difluoro‐3‐oxo‐2,3‐dihydro‐1*H*‐indene‐2,1‐diylidene))dimalononitrile (Y6), a well‐known high‐performance SMA, as the pairing acceptor for both the SMD and GSMDs in OSC fabrication.^[^ ^57^ ^]^

The frontier orbital energy levels of the photoactive materials were analyzed using cyclic voltammetry (CV) (Figure 1b; Figure S14, Supporting Information). All donor materials showed well‐aligned lowest unoccupied molecular orbital (LUMO) and highest occupied molecular orbital (HOMO) energy levels with those of Y6, indicating sufficient driving forces for efficient exciton dissociation at the donor–acceptor interface. Additionally, due to the increased number of electron‐donating BDT units, the two GSMDs exhibited slightly higher LUMO levels (−3.63 to −3.61 eV) compared to **BTR‐Cl** (−3.70 eV), suggesting improved energy alignment with SMA for efficient charge separation (**Table** **1**). To gain further insights into HOMO and LUMO energy levels, density functional theory (DFT) calculations were conducted at B3LYP/6‐31G* (d) level (Figures S15 and S16, Supporting Information).^[^ ^58^, ^59^ ^]^ The HOMO and LUMO energy levels of **GSMD‐*anti*** were slightly upshifted relative to **GSMD‐*syn***, consistent with the results from CV measurement. Furthermore, the π‐electron density in HOMO of **GSMD‐*anti*** is well‐distributed across the entire donor backbone, whereas that of **GSMD‐*syn*** shows electron density localized primarily on BDT core. This extended delocalization in **GSMD‐*anti*** likely enhances efficient charge transport by broadening the effective conjugation length and promoting greater intermolecular overlap.^[^ ^60^, ^61^ ^]^

**Table 1:** Material properties of donors used in this study.

Donor	*E*_g_^opt,^ ^a^ [eV]	*E*_HOMO_ ^b^ [eV]	*E*_LUMO_ ^c^ [eV]	*λ*_max_^sol^ [nm]	*λ*_max_^film^ [nm]	*L*_c(010)_^IP,^ ^d^ [nm]
BTR‐Cl	1.76	−5.46	−3.70	515	577	5.9
GSMD‐*syn*	1.84	−5.47	−3.63	508	558	3.9
GSMD‐*anti*	1.84	−5.45	−3.61	511	562	4.8

^a^ Obtained from the absorption onsets in thin films from chloroform solution using *E*_g_^opt^ = 1240/*λ*_film_^edge^.

^b^ Measured by CV.

^c^ *E*_LUMO_ = *E*_HOMO_ + *E*_g_^opt^.

^d^ Estimated from IP (010) scattering peaks (*q*_xy_ ≈ 1.7 Å^−1^) in the GIXS profiles.

The optical properties of **BTR‐Cl** and the two GSMDs were investigated using ultraviolet–visible (UV–vis) absorption spectroscopy (Figure 1c). All donor materials showed maximum absorption wavelength (*λ*_max_) in the range of 558–577 nm, complementing the absorption of the Y6 SMA (*λ*_max_ = 825 nm). Notably, absorption spectra of the two GSMDs exhibited a blue‐shift (*λ*_max_ = 558–562 nm) compared to **BTR‐Cl** (*λ*_max_ = 577 nm), thereby enhancing their complementary absorption with Y6 and promoting efficient charge generation in OSCs. In addition, despite having the same backbone structure, **GSMD**‐***anti*** showed a slightly red‐shifted absorption (*λ*_max_ = 562 nm) compared to **GSMD**‐***syn*** (*λ*_max_ = 558 nm). This suggests that **GSMD**‐***anti*** has a stronger aggregation tendency than **GSMD**‐***syn***.

To further understand the effect of side‐chain orientation on the backbone planarity of GSMDs, we performed DFT calculations to obtain their optimized molecular geometries (Figure 1e; Figure S17, Supporting Information). The results revealed that **GSMD**‐***anti***, with oppositely oriented alkyl side chains, exhibited markedly improved backbone planarity compared to **GSMD**‐***syn***, which has side chains aligned in the same direction. Specifically, the torsional angles between the thiophene units in **GSMD**‐***anti*** and **GSMD**‐***syn*** were determined to be 25.0° and 51.5°, respectively.

Differential scanning calorimetry (DSC) measurements were conducted to investigate the crystallinity of the donor materials (Figure S18 and Table S1, Supporting Information). To preserve the thermal history imparted by solvent processing, pristine donor solutions were spin‐coated onto glass substrates. The as‐cast films were then directly transferred to DSC pans and subjected to first heating scans for thermal analysis.^[^ ^62^ ^]^ The reference **BTR‐Cl** SMD exhibited two distinct melting temperatures (*T*_m_s) of 202.3 and 227.1 °C in its heating profile, indicative of strong crystallinity and liquid crystalline behavior.^[^ ^18^ ^]^ In contrast, the GSMDs did not show melting transitions but exhibited cold‐crystallization peaks near 250 °C, corresponding to the onset of reorganization and recrystallization of imperfect crystals.^[^ ^63^, ^64^, ^65^ ^]^ These results suggest that both GSMDs possess lower crystallinity than **BTR‐Cl**, likely due to their larger molecular sizes and reduced self‐assembly characteristics.^[^ ^66^ ^]^ Between the two GSMDs, **GSMD**‐***anti*** exhibited a significantly lower cold‐crystallization enthalpy (∆*H*_cc_ = 65.5 J g^−1^) compared to **GSMD**‐***syn*** (100.1 J g^−1^). This indicates that **GSMD**‐***syn*** contains a greater proportion of amorphous domains and disordered crystals, while **GSMD**‐***anti*** exhibits a higher degree of crystallinity and a larger fraction of ordered crystalline domains.

These results were further corroborated by thin‐film crystallinity analysis using grazing‐incidence X‐ray scattering (GIXS) measurements (Figure 1d; Figure S19, Supporting Information). When comparing coherence lengths (*L*_c_s) of (010) scattering peaks in the in‐plane (IP) direction, **BTR‐Cl** showed a higher *L*_c(010)_^IP^ of 5.9 nm compared to 3.9–4.8 nm for the GSMDs. In addition, **GSMD**‐***anti*** exhibited a higher *L*_c(010)_^IP^ of 4.8 nm compared to **GSMD**‐***syn*** (*L*_c(010)_^IP^ = 3.9 nm), despite their identical backbone structures. Charge transport properties were further evaluated using space‐charge‐limited current (SCLC) measurements (Table S1, Supporting Information).^[^ ^67^ ^]^ Although all donor materials exhibited high hole mobilities (*µ*_h_s) exceeding 10^−4^ cm^2^ V^−1^ s^−1^, their relative mobilities were consistent with their crystallinity trends: **GSMD**‐***syn*** (1.6 × 10^−4^ cm^2^ V^−1^ s^−1^) < **GSMD**‐***anti*** (2.0 × 10^−4^ cm^2^ V^−1^ s^−1^) < **BTR‐Cl** (2.9 × 10^−4^ cm^2^ V^−1^ s^−1^).

Overall, the GSMDs demonstrated lower aggregating and crystalline tendency than **BTR‐Cl**, attributable to their increased molecular size. However, between the two GSMDs, **GSMD**‐***anti***, featuring oppositely aligned alkyl side chains, exhibited significantly lower steric hindrance along the backbone than **GSMD**‐***syn***. This enabled greater backbone planarity, higher crystallinity, and improved charge transport relative to **GSMD**‐***syn***.

### 2.2 Photovoltaic Properties

**Figure 2:**
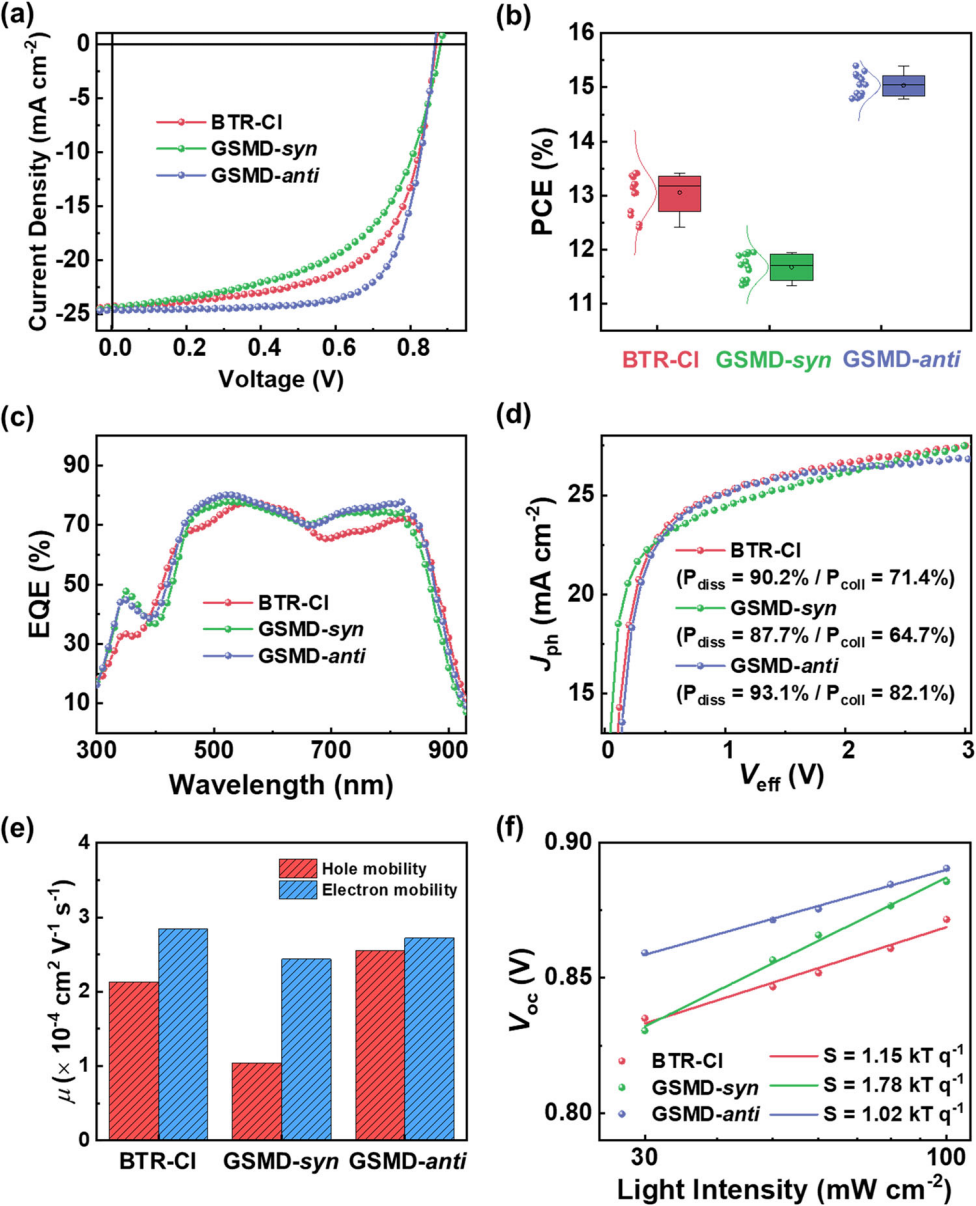
a) *J*–*V* curves, b) Gaussian function fitted PCE distribution, c) EQE spectra, d) *J*_ph_–*V*_eff_ curves, e) SCLC mobilities, and f) light intensity‐dependent *V*_oc_ plot of the OSCs based on BTR‐Cl and GSMDs.

To evaluate the photovoltaic performance of the different donor materials, OSCs with conventional architectures were fabricated (**Figure** **2**). Detailed fabrication procedures are provided in the Supporting Information. The current density–voltage (*J*–*V*) characteristics and the corresponding photovoltaic parameters are presented in Figure 2a and **Table** **2**, respectively. The OSCs based on the **BTR‐Cl** SMD showed a moderate PCE of 13.41%, with an open circuit voltage (*V*_oc_) of 0.87 V, a short‐circuit current density (*J*_sc_) of 24.25 mA cm^−2^, and a fill factor (FF) of 0.64. These results are consistent with previously reported performances of **BTR‐Cl**:Y6 blend‐based devices.^[^ ^18^, ^68^ ^]^ Interestingly, the OSCs based on the two different GSMDs showed markedly different photovoltaic performances. For instance, the **GSMD**‐***syn***:Y6‐based devices exhibited a lower PCE of 11.95% compared to **BTR‐Cl**:Y6, whereas the **GSMD**‐***anti***:Y6‐based devices achieved an even higher PCE of 15.40%. These performance differences were primarily driven by variations in FF. Specifically, while the *V*_oc_ (0.87–0.88 V) remained comparable across all three devices, the FF followed the trend: **GSMD**‐***syn***:Y6 (0.56) < **BTR‐Cl**:Y6 (0.64) < **GSMD**‐***anti***:Y6 (0.72). In addition, although the variation was relatively modest, the **GSMD**‐***anti***:Y6 device exhibited the highest *J*_sc_ (24.54 mA cm^−2^) among the systems evaluated.

**Table 2:** Photovoltaic performance of BTR‐Cl and GSMDs‐based OSCs.

Active material (Y6‐based)	*V*_oc_ [V]	*J*_sc_ [mA cm^−2^]	Calc. *J*_sc_ ^a^ [mA cm^−2^]	FF	PCE_max_ (PCE_avg_)^b^ [%]
BTR‐Cl	0.87 (0.88 ± 0.01)	24.25 (23.47 ± 0.92)	23.08	0.64 (0.64 ± 0.02)	13.41 (13.06 ± 0.36)
GSMD‐*syn*	0.88 (0.88 ± 0.01)	24.35 (24.06 ± 0.34)	23.20	0.56 (0.55 ± 0.01)	11.95 (11.67 ± 0.24)
GSMD‐*anti*	0.87 (0.87 ± 0.01)	24.54 (24.20 ± 0.55)	24.01	0.72 (0.68 ± 0.02)	15.40 (15.05 ± 0.20)

^a^ Calculated from EQE spectra.

^b^ Average values obtained from at least 10 independent devices.

The PCE distributions of all devices were fitted well to Gaussian functions, indicating high reproducibility of the OSCs (Figure 2b). The external quantum efficiency (EQE) spectra in all OSCs exhibited efficient charge generation throughout a wide wavelength range (300–900 nm), supporting their high *J*_sc_s (> 24 mA cm^−2^) (Figure 2c). In addition, calculated *J*_sc_ values from the EQE spectra were well‐matched with the device *J*_sc_s within a 5% error range (Table 2). The EQE enhancement between 450−550 nm in the **GSMD‐*anti***‐based devices contributed to the increase in *J*_sc_.

To further elucidate the origins of different photovoltaic properties, we evaluated the charge generation, transport, and recombination behaviors of the OSCs. The charge generation properties were assessed by measuring photocurrent density (*J*_ph_) at effective voltage (*V*_eff_) (Figure 2d).^[^ ^69^ ^]^ Among the different systems, the **GSMD**‐***anti***:Y6‐based OSCs showed the highest exciton dissociation probability (*P*_diss_) of 93.1% and charge collection probability (*P*_coll_) of 82.1%, outperforming both the **BTR‐Cl**:Y6 (*P*_diss_ = 90.2% and *P*_coll_ = 71.4%) and **GSMD**‐***syn***:Y6 (*P*_diss_ = 87.7% and *P*_coll_ = 64.7%). Consequently, in the **GSMD‐*anti***:Y6 OSCs, a greater fraction of excitons was efficiently dissociated and converted into free charge carriers, which were subsequently well‐collected at the electrodes. This explains the higher *J*_sc_ observed in the **GSMD‐*anti***:Y6 system, compared to the two other systems.

Charge transport properties of the OSCs were evaluated by measuring the charge carrier mobilities using the SCLC method (Figure 2e; Table S2, Supporting Information).^[^ ^67^ ^]^ The electron mobility (*µ*_e_) values were similar across all blends, ranging from 2.4 to 2.8 × 10^−4^ cm^2^ V^−1^ s^−1^, as the same Y6 SMA was used. Meanwhile, the *µ*_h_s of **GSMD**‐***anti***:Y6 and **BTR‐Cl**:Y6 (2.1–2.6 × 10^−4^ cm^2^ V^−1^ s^−1^) were at least twice as high as that of **GSMD**‐***syn***:Y6 (*µ*_h_ = 1.0 × 10^−4^ cm^2^ V^−1^ s^−1^). As a result, *µ*_e_/*µ*_h_ ratios of **GSMD**‐***anti***:Y6 (1.1) and **BTR‐Cl**:Y6 (1.3) were more balanced compared to the significantly unbalanced ratio observed for **GSMD**‐***syn***:Y6 (2.3).

Charge recombination properties were analyzed by measuring dependence of *V*_oc_ and *J*_sc_ on light intensity (*P*) of the OSCs (Figure 2f; Figure S20, Supporting Information).^[^ ^70^ ^]^ In *V*_oc_ versus *P* plot, the slope (*S*) approaches 1 *k*_B_T *q* ^−1^ (where *k*_B_ = Boltzmann constant, T = temperature in Kelvin, and *q* = elementary charge) as the extent of monomolecular or trap‐assisted recombination decreases. The **GSMD**‐***anti***:Y6‐based OSCs exhibited an *S* value of 1.02 *k*_B_T *q* ^−1^, much closer to the ideal value of unity compared to the other devices (*S* = 1.15−1.78 *k*_B_T *q* ^−1^), indicating suppressed monomolecular or trap‐assisted recombination. In particular, the **GSMD**‐***syn***:Y6‐based OSCs showed a significantly higher *S* value of 1.78 *k*_B_T *q* ^−1^, suggesting severe monomolecular or trap‐assisted recombination in these devices. On the other hand, the slope (α) in the *J*_sc_ versus *P* plot reflects the extent of bimolecular recombination (Figure S20, Supporting Information). The α values for all devices ranged from 0.92 to 0.94, indicating comparable levels of bimolecular recombination across all systems. Overall, the **GSMD**‐***anti***:Y6‐based OSCs exhibited superior charge generation compared to the other devices, accounting for their higher *J*_sc_. In addition, the more balanced charge transport and reduced monomolecular or trap‐assisted recombination in these devices explain their significantly higher FF relative to the other systems.

### 2.3 Operational Stability Under 1‐Sun Illumination

**Figure 3:**
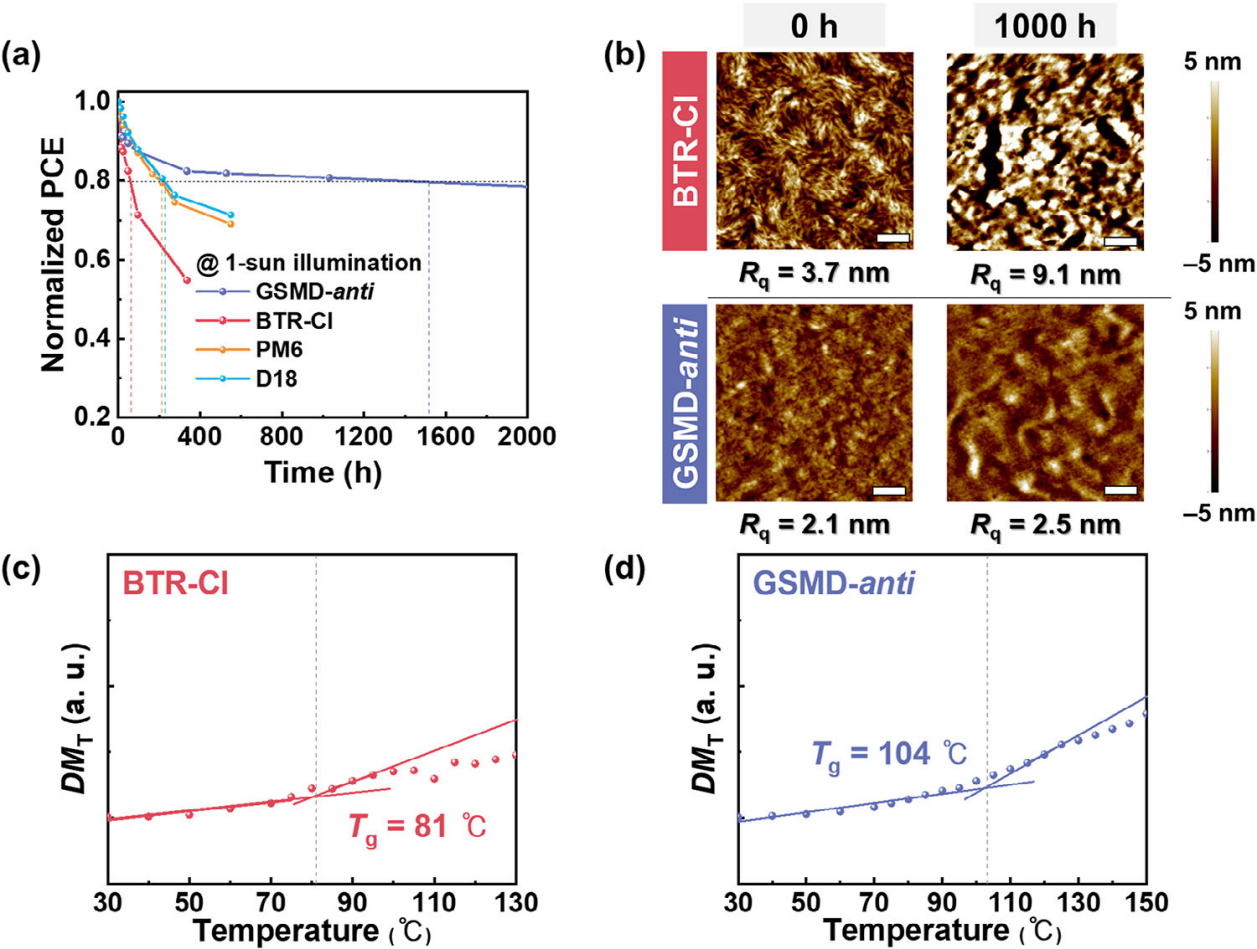
a) Normalized PCE values under 1‐sun illumination. b) AFM height images of blend films (active layers) before and after 1000 h of illumination (scale bars are 500 nm). c–d) *DM*_T_ plots of (c) BTR‐Cl and (d) GSMD‐*anti* films as a function of temperature.

Operational stability is another essential requirement for the commercialization of OSCs. To evaluate the photostability of our devices, we monitored performance degradation under continuous 1‐sun illumination (100 mW cm^−2^) (**Figure** **3**). Detailed experimental conditions for the photostability tests are provided in the Supporting Information. The *t*_80%_ lifetime, defined as the time required for the PCE to drop to 80% of its initial value, was determined by linear extrapolation after measuring device stability up to 1000 h.^[^ ^71^, ^72^ ^]^ Remarkably, the **GSMD‐*anti***:Y6‐based OSCs demonstrated remarkably enhanced photostability compared to the **BTR‐Cl**:Y6‐based devices. Specifically, the *t*_80%_ lifetimes of **BTR‐Cl**:Y6 and **GSMD‐*anti***:Y6 were 60 and 1510 h, respectively (Figure 3a). Furthermore, the **GSMD‐*anti***:Y6 devices demonstrated higher *t*_80%_ lifetime compared to the OSCs based on conventional high‐performance *P*_D_s such as PM6 and D18 (*t*_80%_ lifetime < 400 h), highlighting the advantages of GSMD as promising photoactive materials for stable OSCs.^[^ ^73^, ^74^ ^]^

One of the most critical factors influencing OSC stability is the change in blend morphology under light exposure and thermal stress. To understand the high photostability of the **GSMD‐*anti***:Y6‐based OSCs, we investigated the blend morphologies under optimal device conditions and after prolonged light exposure using atomic force microscopy (AFM) (Figure 3b). AFM height images revealed significant morphological changes in the **BTR‐Cl**:Y6 blend after 1000 h of light exposure. For example, its surface roughness (*R*_q_) increased from 3.7 to 9.1 nm, accompanied by the formation of larger, coarsened domains with sharper boundaries. In contrast, the **GSMD‐*anti***:Y6 blend maintained a stable morphology, as evidenced by its nearly unchanged *R*_q_ before and after light exposure (from 2.1 to 2.5 nm).

The morphological stability is closely linked to the thermal diffusion characteristics of the donor materials, for which *T*_g_ serves as a crucial parameter.^[^ ^29^ ^]^ *T_g_* denotes the temperature at which the material changes from a rigid to a more mobile state, facilitating molecular rearrangement within imperfect crystalline regions. To elucidate the mechanism underlying the enhanced morphological stability observed in **GSMD‐*anti***:Y6 OSCs, we determined the *T*_g_ values of the **GSMD‐*anti*** and **BTR‐Cl** by tracking variations in UV–vis absorbance as a function of annealing temperature (Figures 3c,d; Figure S21, Supporting Information).^[^ ^28^, ^75^ ^]^ A deviation metric (*DM*_T_), which quantifies changes in the absorbance during the annealing process, was employed to estimate the *T*_g_s (see Supporting Information for further information).^[^ ^76^ ^]^ The *T*_g_ of **GSMD‐*anti*** was measured to be 104 °C, which was higher than that of **BTR‐Cl** (*T*_g_ = 81 °C). This *T*_g_ value of **BTR‐Cl** agrees well with the previously reported value.^[^ ^77^ ^]^ This suggests that the onset temperature for thermally induced molecular motion and diffusion in the **GSMD‐*anti*** film is substantially higher than that in the **BTR‐Cl**. Moreover, the significantly higher *T*_g_ of **GSMD‐*anti*** indicates markedly slower diffusion kinetics, considering that the diffusion coefficient of donors exponentially decreases with increasing *T*_g_ at given acceptor pairs.^[^ ^28^, ^29^ ^]^ This elevated *T*_g_ is likely due to the larger molecular size of **GSMD‐*anti*** compared to **BTR‐Cl**.^[^ ^35^, ^78^ ^]^ The reduced molecular diffusion effectively suppresses phase separation during prolonged illumination, which explains the significantly enhanced photostability of the **GSMD‐*anti***:Y6 devices. Similarly, the **GSMD‐*syn*** also exhibited a higher *T*_g_ (103 °C) compared to that of **BTR‐Cl**, which accounts for its improved photostability in the OSC compared to the **BTR‐Cl**‐based OSC (Figures S21 and S22, Supporting Information).

### 2.4 Morphological Properties

**Figure 4:**
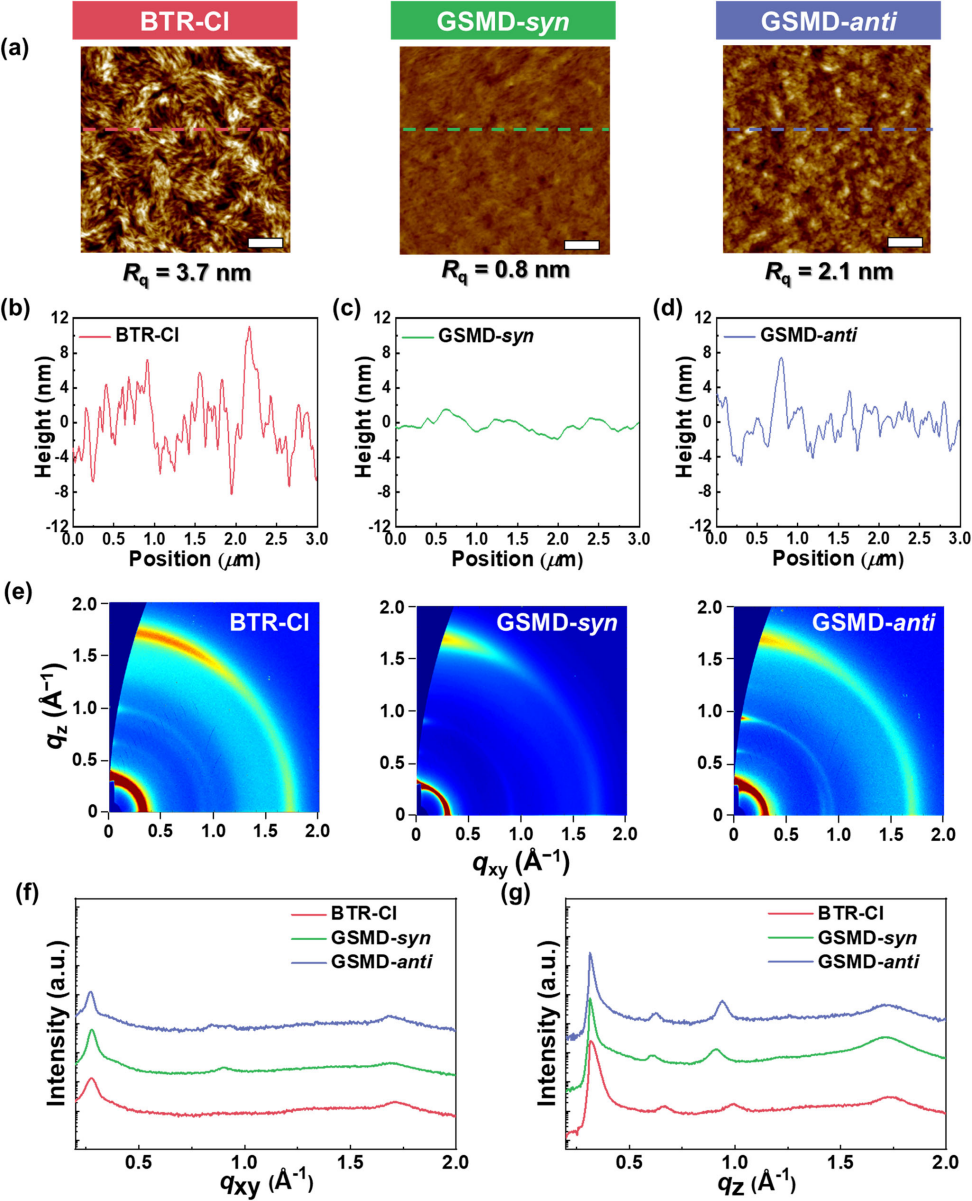
a) AFM height images of donor:Y6 blend films (scale bars are 500 nm). b–d) Height profiles of (b) BTR‐Cl:Y6, (c) GSMD‐*syn*:Y6, and (d) GSMD‐*anti*:Y6 blend films obtained from AFM height images. e) GIXS 2D‐images and f,g) line‐cut profiles of the donor:Y6 blend films in the (f) IP and (g) OOP directions.

To further investigate the differences in electrical and photovoltaic properties depending on the donor types, we analyzed the morphological characteristics of the donor:Y6 blend films. The surface morphologies were examined by measuring AFM height images (**Figures** **4**a–d). Both **BTR‐Cl**:Y6 and **GSMD‐*anti***:Y6 blends exhibited fibrillar structures, indicative of connected charge transport pathways. In contrast, the **GSMD‐*syn***:Y6 blend did not exhibit discernible fibrillar features and instead showed a relatively uniform morphology. Notably, the **BTR‐Cl**:Y6 blend showed larger grain sizes, a rougher surface, and more prominent aggregations compared to the GSMDs:Y6 blends. For instance, the *R*_q_ of **BTR‐Cl**:Y6 (3.7 nm) was higher compared to the **GSMD‐*syn***:Y6 (0.8 nm) and **GSMD‐*anti***:Y6 (2.1 nm). The pronounced phase separation observed in **BTR‐Cl**:Y6 blends is presumably attributed to crystallization‐driven phase separation, induced by the strong self‐assembly characteristics of the **BTR‐Cl**, as evidenced by the DSC and pristine film GIXS results (Figure 1d; Figure S18, Supporting Information).^[^ ^17^, ^18^ ^]^ The increased phase separation and presence of rough aggregations in **BTR‐Cl**:Y6 blends could serve as trap sites for free charge carriers, potentially leading to increased charge recombination in OSCs.

The crystalline structures of the donor:Y6 blend films were analyzed using GIXS (Figures 4e–g). All the blend films exhibited face‐on preferential packing structures, beneficial for vertical charge transport. To quantify the relative crystallinity, the *L*_c_ values corresponding to the (010) π–π stacking peaks in the out‐of‐plane (OOP) direction were estimated (**Table** **3**). The *L*_c(010)_ values increased in the order of **GSMD‐*syn***:Y6 (3.2 nm) < **GSMD‐*anti***:Y6 (4.0 nm) < **BTR‐Cl**:Y6 (4.7 nm), indicating a lower degree of crystallinity in the **GSMD‐*syn***:Y6 blend compared to the others. This reduced crystallinity in the **GSMD‐*syn***:Y6 blend is consistent with the absence of fibrillar structures observed in AFM images. Internal morphology analysis using resonant soft X‐ray scattering (RSoXS) further supported these observations (Figure S23, Supporting Information). In line with the AFM and GIXS results, **BTR‐Cl**:Y6 exhibited significantly higher domain spacing (*d*_RSoXS_ = 233 nm) and relative domain purity (*r*‐DP = 1.00) compared to the **GSMD‐*syn***:Y6 (*d*_RSoXS_ = 37 nm and *r*‐DP = 0.78) and **GSMD‐*anti***:Y6 (*d*_RSoXS_ = 48 nm and *r*‐DP = 0.86) blend films. This result indicates more pronounced phase separation and the formation of purer domains in the **BTR‐Cl**:Y6 blend compared to GSMDs:Y6 blend films. Between GSMDs, the lower *r*‐DP value of **GSMD‐*syn***:Y6 (0.78) compared to that of **GSMD‐*anti***:Y6 (*r*‐DP = 0.86) suggests the presence of less‐pure and disordered domains.

**Table 3:** Morphological properties of donor:Y6 blend films.

System	*R*_q_ [nm]	*L*_c(010)_ ^a^ [nm]	*d*_RSoXS_ ^b^ [nm]	*r*‐DP^b^
BTR‐Cl:Y6	3.7	4.7	233	1.00
GSMD‐*syn*:Y6	0.8	3.2	37	0.78
GSMD‐*anti*:Y6	2.1	4.0	48	0.86

^a^ Estimated from the GIXS profiles of donor:Y6 blend along the OOP direction.

^b^ Estimated from RSoXS profiles at 284.8 eV.

Based on combined morphological analyses, the **BTR‐Cl**:Y6 blend exhibited distinct fibrillar structures conducive to efficient charge transport. However, it also showed pronounced phase separation and aggregation, characterized by excessively large domain sizes. These features are attributed to the strong self‐assembling behavior and high crystallinity of **BTR‐Cl**. As a result, although the **BTR‐Cl**:Y6‐based OSCs exhibited high charge mobility, they also suffered from increased charge recombination, leading to a moderate PCE of 13.4%. In contrast, the **GSMD‐*syn***:Y6 blend displayed a more intermixed morphology but lacked fibrillar networks. This was supported by the lowest values of *R*_q_, *L*_c(010)_, and *r*‐DP values among the tested systems. Consequently, **GSMD‐*syn***:Y6‐based OSCs exhibited limited charge transport and a comparatively low PCE of 11.9%. Meanwhile, the **GSMD‐*anti***:Y6 blend exhibited an optimal intermediate morphology between the two other systems. Despite having the same backbone structure and size as **GSMD‐*syn***, **GSMD‐*anti***:Y6 formed well‐connected fibrillar networks that supported efficient charge transport. This enhancement is attributed to the anti‐oriented side chains in **GSMD‐*anti***, which reduced steric hindrance, thereby promoting higher aggregation and crystallinity compared to **GSMD‐*syn***. At the same time, the **GSMD‐*anti***:Y6 blend showed a more finely intermixed morphology with less pronounced aggregation than the **BTR‐Cl**:Y6 blend. This is likely due to the reduced self‐assembly tendency of **GSMD‐*anti***, which stems from its larger molecular size compared to **BTR‐Cl**. Together, these favorable morphological features enabled both efficient charge transport and suppressed recombination. As a result, the **GSMD‐*anti***:Y6 system simultaneously achieved high PCE (15.4%) and excellent photostability (*t*_80%_ lifetime > 1500 h).

### 2.5 Photovoltaic Performance of Ternary System

**Figure 5:**
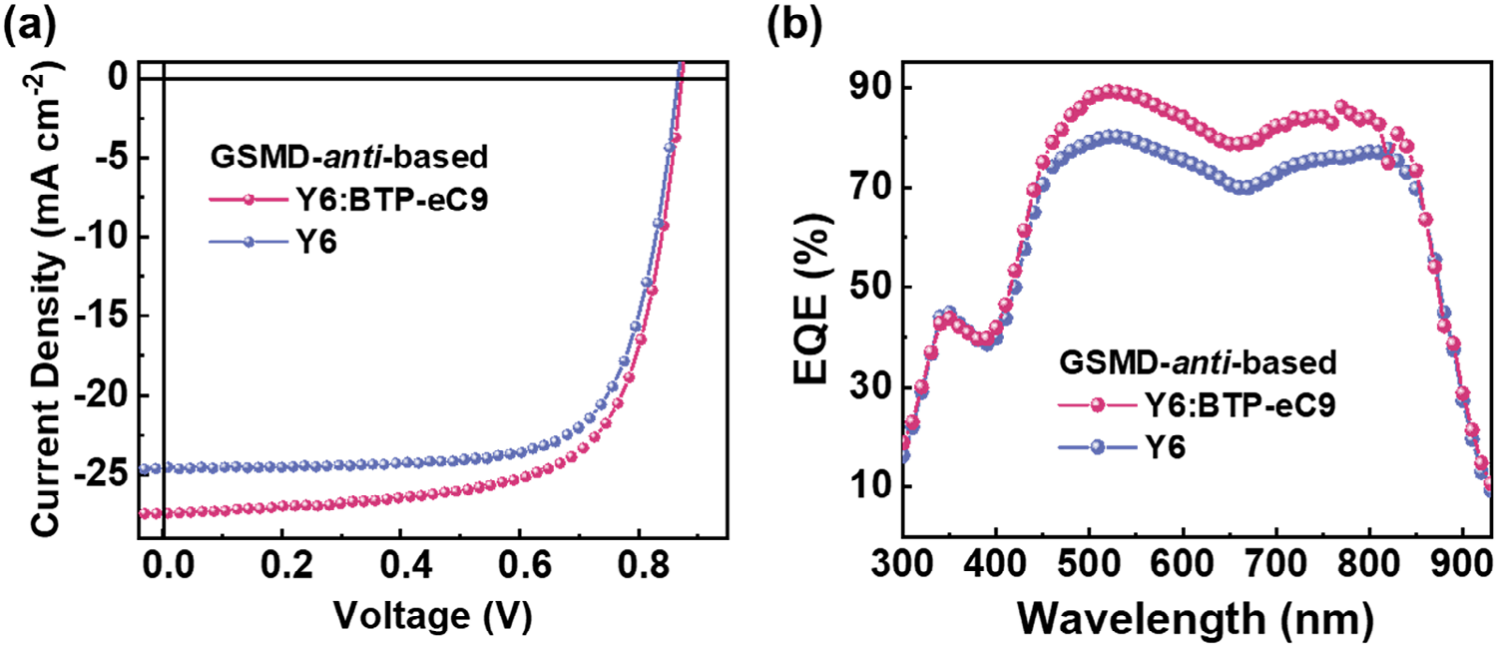
a) *J*–*V* curves and b) EQE spectra of OSCs based on the GSMD‐*anti*:Y6 binary and GSMD‐*anti*:Y6:BTP‐eC9 ternary systems.

To further explore the potential of the developed **GSMD‐*anti*** donor, we fabricated ternary OSCs by incorporating a third SMA component (BTP‐eC9) into the **GSMD‐*anti***:Y6 photoactive system (**Figure** **5** and **Table** **4**). BTP‐eC9 complements the near‐infrared (NIR) absorption region of Y6 and forms cascade energy levels with both **GSMD‐*anti*** and Y6, thereby facilitating more efficient charge generation (Figures S24 and S25, Supporting Information).^[^ ^79^ ^]^ In detail, BTP‐eC9 exhibits a slightly up‐shifted LUMO (–4.29 eV) compared to Y6 (–4.32 eV), resulting in the LUMO level between those of **GSMD‐*anti*** and Y6 (Figure S26, Supporting Information).^[^ ^80^ ^]^ The resulting ternary OSCs (**GSMD‐*anti***:Y6:BTP‐eC9) achieved an enhanced PCE of 16.50%, compared to 15.40% for the binary **GSMD‐*anti***:Y6 devices (Figure 5a). This improvement in photovoltaic performance was primarily due to an increase in *J*_sc_, which is supported by EQE responses across a broad absorption range (300–900 nm) (Figure 5b). For example, the complementary absorption of BTP‐eC9 in the NIR region of 800–900 nm resulted in an additional EQE enhancement. To the best of our knowledge, this represents one of the highest PCEs reported for GSMD‐based OSCs, underscoring the potential of **GSMD‐*anti*** as promising photoactive donor candidates. And, the ternary **GSMD‐*anti***:Y6:BTP‐eC9 device showed comparable photostability (*t*_80%_ = 1470 h) to binary **GSMD‐*anti***:Y6 photostability (*t*_80%_ = 1510 h) (Figure S27, Supporting Information). It is speculated that this result could be attributed to the formation of alloy‐like phase between BTP‐eC9 and Y6, owing to their structural similarity, producing a similar blend morphology as the **GSMD‐*anti***:Y6 blend.^[^ ^81^, ^82^ ^]^ Overall, these results demonstrate that the structural benefits of GSMD contribute to the OSC stability. For the further advancement of GSMD‐based systems, it is crucial to design new GSMDs with optimized linker structures that enhance charge transport properties and blend morphology with the acceptor materials. Additionally, future research should focus on developing GSMDs that further increase device performance while simplifying the synthesis route, thus reducing production complexity and cost for the GSMDs.^[^ ^23^, ^60^ ^]^

**Table 4:** Photovoltaic performance of GSMD‐*anti*:Y6:BTP‐eC9 system.

Blend System	*V*_oc_ [V]	*J*_sc_ [mA cm^−2^]	FF	PCE_max_ (PCE_avg_)^b^ [%]
GSMD‐*anti*:Y6:BTP‐eC9^a^	0.87 (0.88 ± 0.00)	27.45 (26.85 ± 0.44)	0.69 (0.68 ± 0.01)	16.50 (15.87 ± 0.35)

^a^ The ternary OSCs were fabricated using a 1.5:0.7:0.3 weight ratio and Br‐2PACz as the hole transport layer.

^b^ Average value obtained from at least 10 independent devices.

## 3 Conclusion

In summary, we synthesized new GSMDs with controlled backbone planarity and successfully developed efficient (PCE > 15%) and photostable (*t*_80%_ lifetime > 1500 h) OSCs. To optimize the crystallinity and charge transport properties of the GSMDs, we systematically tuned their backbone planarity by controlling the orientation of alkyl side chains. Specifically, we synthesized two GSMDs with distinct molecular configurations: 1) **GSMD‐*syn***, in which the side chains are aligned in the same direction, and 2) **GSMD‐*anti***, in which the side chains are aligned in the opposite direction. The anti‐oriented side chain configuration effectively minimizes steric hindrance between adjacent backbone units, leading to enhanced crystallinity and charge transport in the active layer. As a result, OSCs based on **GSMD‐*anti***:Y6 achieved a high PCE of 15.4%, significantly outperforming those based on the conventional SMD (**BTR‐Cl**:Y6, PCE = 13.4%) and **GSMD‐*syn***:Y6 (PCE = 11.9%). Notably, **GSMD‐*anti***:Y6 devices also exhibited significantly enhanced photostability (*t*_80%_ lifetime = 1510 h), compared to **BTR‐Cl**:Y6 (*t*_80%_ lifetime = 60 h), which is attributed to reduced molecular diffusion resulting from the larger molecular size of **GSMD‐*anti***. Furthermore, the PCE value of the **GSMD‐*anti***‐based OSCs was improved to 16.5% by constructing all‐small‐molecule ternary blend, while maintaining comparable photostability (1470 h) to the binary system. This work offers a useful design strategy for developing efficient, stable GSMD‐based OSCs.

## Conflict of Interest

The authors declare no conflict of interest.

## Supporting information



Supporting Information

Supporting Information

## Data Availability

The data that support the findings of this study are available from the corresponding author upon reasonable request.
