# Supplementary material for: Giant Small‐Molecule Donors With Controlled Backbone Planarity Afford High‐Performance and Photostable Organic Solar Cells
Source: Adv Sci (Weinh). 2025 Nov 19;13(7):e12427. doi: 10.1002/advs.202512427 (PMC12866850; doi:10.1002/advs.202512427)
Supplement: Supplementary file 1 — Supporting Information [file ADVS-13-e12427-s002.pdf]

Supporting Information for

Giant Small-Molecule Donors with Controlled Backbone Planarity Afford High-Performance and Photostable Organic Solar Cells

Hyerin Jeon[†,a], *Seunghoon Song*[†,b], *Jin-Woo Lee*[a], *Yun-Hi Kim*[b,*], *and Bumjoon J. Kim*[a,*]

[a] Department of Chemical and Biomolecular Engineering, Korea Advanced Institute of Science and Technology (KAIST), Daejeon 34141, Republic of Korea
E-mail: bumjoonkim@kaist.ac.kr

[b] Department of Chemistry and RIMA, Gyeongsang National University, Jinju 528258, Republic of Korea
E-mail: ykim@gnu.ac.kr

[†]These authors contributed equally to this work.

Supporting Schemes, Figures, and Tables

Scheme S1. Synthetic scheme for GSMD-*syn*.

Scheme S2. Synthetic scheme for GSMD-*anti*.

Figure S1. ^{1}H NMR spectrum of compound 1-2.

Figure S2. ^{13}C NMR spectrum of compound 1-2.

Figure S3. ^{1}H NMR spectrum of compound 1-3.

Figure S4. ^{13}C NMR spectrum of compound 1-3.

Figure S5. ^{1}H NMR spectrum of GSMD-*syn*.

Figure S6. MALDI-ToF spectrum of GSMD-*syn*.

Figure S7. ^{1}H NMR spectrum of compound 2-2.

Figure S8. ^{13}C NMR spectrum of compound 2-2.

Figure S9. ^{1}H NMR spectrum of compound 2-3.

Figure S10. ^{13}C NMR spectrum of compound 2-3.

Figure S11. ^{1}H NMR spectrum of GSMD-*anti*.

Figure S12. MALDI-ToF spectrum of GSMD-*anti*.

Figure S13. TGA spectra of BTR-Cl and GSMDs.

Figure S14. CVs of (a) BTR-Cl, (b) GSMD-*syn*, (c) GSMD-*anti*, (d) Y6, and (e) ferrocene.

Figure S15. The frontier molecular orbitals (HOMO and LUMO) from GSMD-*syn* calculated based on DFT simulations.

Figure S16. The frontier molecular orbitals (HOMO and LUMO) from GSMD-*anti* calculated based on DFT simulations.

Figure S17. Optimized molecular conformations of GSMDs; (a) GSMD-*syn* and (b) GSMD-*anti* obtained from DFT simulations.

Figure S18. DSC thermograms of pristine donor films obtained during the 1st heating cycle at a rate of 10 °C min^{-1}.

Figure S19. GIXS line-cut profiles in the out-of-plane direction of the pristine donors.

Figure S20. Light intensity-dependent J_{sc} plots of Y6-based OSCs.

Figure S21. DM_T plot of GSMD-*syn* films as a function of temperature.

Figure S22. Normalized PCE values of binary blend systems under 1-sun illumination.

Figure S23. RSoXS profiles of blend films.
Figure S24. Chemical structures of BTP-eC9.
Figure S25. UV−vis absorption spectra in film of GSMD-*anti*-based blends.
Figure S26. (a) CV of BTP-eC9 and (b) energy level alignment in film of donor and acceptors.
Figure S27. Normalized PCE values under 1-sun illumination.

Table S1. DSC results and SCLC hole mobilities of the pristine donor films.
Table S2. SCLC mobilities for the donors:Y6 blend films.

Experimental Section

Materials**:** All solvents were purchased from Sigma Aldrich, Alfa Aesar, and TCI. All reactions were carried out in a nitrogen atomsphere. All catalysts were purchased from Umicore. 2,6-Dibromo-4,8-bis(4-chloro-5-(2-ethylhexyl)thiophen-2-yl)benzo[1,2-*b*:4,5-*b*']dithiophene, (4,8-bis(4-chloro-5-(2-ethylhexyl)thiophen-2-yl)benzo[1,2-*b*:4,5-*b*']dithiophene-2,6-diyl)bis(trimethylstannane), 3',3''-dihexyl-[2,2':5',2''-terthiophene]-5-carbaldehyde, 3,3''-dioctyl-5''-(tributylstannyl)-[2,2':5',2''-terthiophene]-5-carbaldehyde, 3-hexyl-2-thioxothiazolidin-4-one were synthesized using the similar method as previous reports, respectively.[1] 2,2'-((2*Z*,2'*Z*)-((12,13-Bis(2-ethylhexyl)-3,9-diundecyl-12,13-dihydro-[1,2,5]thiadiazolo[3,4-*e*]thieno[2'',3'':4',5']thieno[2',3':4,5]pyrrolo[3,2-*g*]thieno[2',3':4,5]thieno[3,2-*b*]indole-2,10-diyl)bis(methanylylidene))bis(5,6-difluoro-3-oxo-2,3-dihydro-1*H*-indene-2,1-diylidene))dimalononitrile (Y6) and 2,2'-[[12,13-bis(2-butyloctyl)-12,13-dihydro-3,9-dinonylbisthieno[2'',3'':4',5']thieno[2',3':4,5]pyrrolo[3,2-*e*:2',3'-*g*][2,1,3]benzothiadiazole-2,10-diyl]bis[methylidyne(5,6-chloro-3-oxo-1*H*-indene-2,1(3*H*)-diylidene)]]bis[propanedinitrile] (BTP-eC9) were purchased from Derthon Optoelectronic Materials Sci. Tech. Co. LTD. Poly[(9,9-bis(3'-((*N*,*N*-dimethyl)-*N*-ethylammonium)propyl)-2,7-fluorene)-*alt*-5,5'-bis(2,2'-thiophene)-2,6-naphthalene-1,4,5,8-tetracaboxylic-*N*,*N*'-di(2-ethylhexyl)imide]dibromide (PNDIT-F3N-Br) was synthesized according to the reported method.[2]

Synthesis of giant small-molecule donors

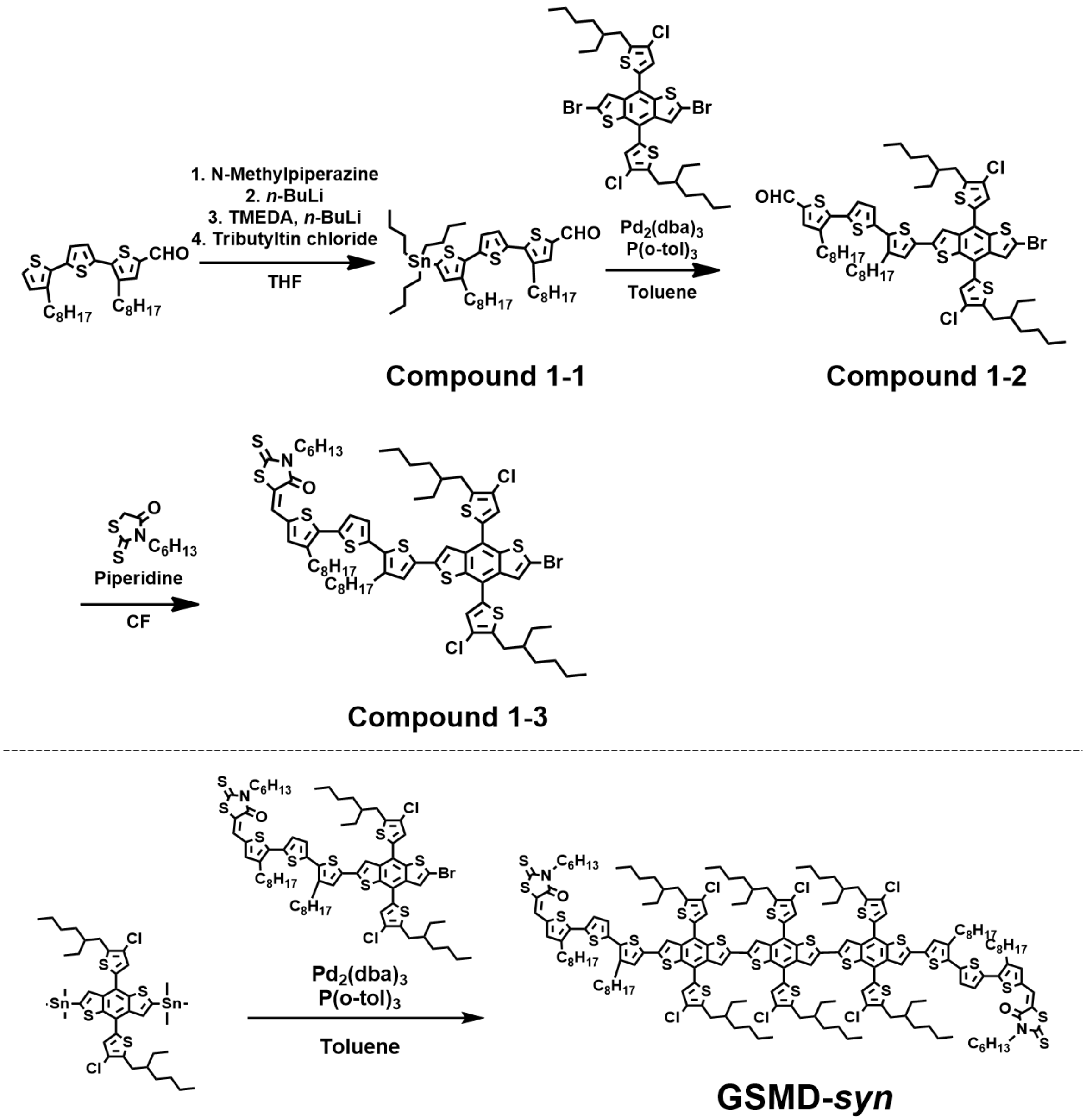

Scheme S1. Synthetic scheme for GSMD-*syn*.

Synthesis of 3,3''-dioctyl-[2,2':5',2''-terthiophene]-5-carbaldehyde (Compound 1-1)

In a 3-necked flask, *N*-methylpiperazine (1.20 g, 12.00 mmol) was dissolved in THF (160 mL) and cooled to −78 °C. *n*-BuLi (2.50 M, 4.80 mL, 12.00 mmol) was slowly added dropwise, followed by stirring for 30 min. Afterward, 3',3''-dioctyl-[2,2':5',2''-terthiophene]-5-carbaldehyde

(5.00 g, 9.98 mmol) was gradually added dropwise and stirred for an additional 30 min. *N*,*N*,*N*',*N*'-Tetrametylethylenediamine (TMEDA) (1.74 g, 14.98 mmol) was then added to the reaction mixture. Subsequently, *n*-BuLi (2.50 M, 6.00 mL, 14.98 mmol) was slowly added dropwise at −78 °C, and the mixture was stirred at a temperature of −70 °C for 2 h. Tributyltin chloride (4.87 g, 14.98 mmol) was added dropwise at −78 °C, and the reaction was followed by stirring for 2 h at room temperature. Then, water was added to the reaction mixture, and the solution was extracted with ether before drying over anhydrous $MgSO_4$. Removing the solvent under reduced pressure gave the crude compound 1-1. Without any further purification, the product was used into the next reaction.

Synthesis of 5''-(6-bromo-4,8-bis(4-chloro-5-(2-ethylhexyl)thiophen-2-yl)benzo[1,2-b:4,5-b']dithiophen-2-yl)-3,3''-dioctyl-[2,2':5',2''-terthiophene]-5-carbaldehyde (Compound 1-2)

Compound 1-1 (6.10 g, 7.57 mmol) and 2,6-dibromo-4,8-bis(4-chloro-5-(2-ethylhexyl)thiophen-2-yl)benzo[1,2-*b*:4,5-*b*']dithiophene (5.98 g, 7.57 mmol) were dissolved in 120 mL toluene with degassing by nitrogen for 30 min. After degassing, $Pd_2(dba)_3$ (0.14 g, 0.15 mmol) and P(*o*-tol)$_3$ (0.18 mg, 0.61 mmol) were added, and the reaction mixture was heated at 110 °C for 12 h. After cooling to room temperature, water was added to the reaction mixture, and the solution was extracted with dichloromethane before drying over anhydrous $MgSO_4$. After removing the organic solvent, the crude product was purified by column chromatography on silica gel with dichloromethane/hexane as an eluent (Yield: 24%). ^{1}H-NMR (CD_2Cl_2, 300MHz): δ (ppm) 9.87 (s, 1H), 7.67-7.65 (d, 2H), 7.62 (s, 1H), 7.33-7.32 (d, 1H), 7.28 (s, 2H), 7.25 (s, 1H), 7.22-7.21 (d, 1H), 2.93-2.81 (m, 8H), 1.83-1.72 (m, 6H), 1.53-1.31 (m, 36H), 1.04-0.88 (m, 18H). ^{13}C-NMR ($CDCl_3$, 400 MHz): 182.54, 141.27, 140.85, 140.48, 140.40, 140.37, 139.02, 138.88, 138.83,

138.56, 138.41, 137.68, 137.05, 136.18, 135.15, 135.08, 134.96, 130.82, 128.83, 128.09, 127.90, 126.47, 125.78, 123.03, 122.95, 122.13, 122.01, 118.40, 117.16, 40.92, 32.55, 32.50, 32.15, 31.87, 30.52, 30.31, 29.64, 29.50, 29.43, 29.25, 28.82, 25.89, 23.05, 22.68, 14.18, 14.12, 10.92, 10.88.

Synthesis of (E)-5-((5''-(6-bromo-4,8-bis(4-chloro-5-(2-ethylhexyl)thiophen-2-yl)benzo[1,2-b:4,5-b']dithiophen-2-yl)-3,3''-dioctyl-[2,2':5',2''-terthiophen]-5-yl)methylene)-3-hexyl-2-thioxothiazolidin-4-one (Compound 1-3)

Compound 1-2 (2.50 g, 2.04 mmol), 3-hexyl-2-thioxothiazolidin-4-one (1.10 g, 0.48 mmol) and piperidine (0.64 g, 8.16 mmol) were dissolved in chloroform (100 mL). The reaction mixture was heated at 60 °C for 8 h. After cooling to room temperature, water was added to the reaction mixture, and the solution was extracted with dichloromethane before drying over anhydrous $MgSO_4$. After removing the organic solvent, the crude product was purified by column chromatography using dichloromethane/hexane as an eluent (Yield: 86%). ^{1}H-NMR (CD_2Cl_2, 300MHz): δ (ppm) 7.82 (s, 1H), 7.65 (s, 1H), 7.62 (s, 1H), 7.32 (s, 1H), 7.31-7.30 (d, 1H), 7.28 (s, 2H), 7.24 (s, 1H), 7.22-7.21(d, 1H), 4.15-4.10 (t, 2H), 2.93-2.82 (m, 8H), 1.83-1.71 (m, 8H), 1.53-1.31 (m, 42H), 1.04-0.88 (m, 21H). ^{13}C-NMR ($CDCl_3$, 400 MHz): 192.25, 167.54, 141.19, 141.14, 140.39, 139.24, 138.88, 138.82, 138.43, 138.06, 137.31, 137.24, 137.06, 136.16, 135.37, 135.09, 135.03, 130.99, 128.85, 128.10, 127.32, 126.46, 125.78, 124.81, 123.03, 122.95, 122.10, 121.99, 120.67, 118.35, 117.13, 44.88, 40.92, 32.55, 32.50, 32.14, 31.90, 3136, 30.49, 30.29, 29.69, 29.65, 29.55, 29.46, 29.43, 29.27, 28.83, 26.96, 26.47, 25.89, 23.06, 22.69, 22.52, 14.19, 14.13, 14.02, 10.93, 10.88.

Synthesis of GSMD-syn

(4,8-Bis(4-chloro-5-(2-ethylhexyl)thiophen-2-yl)benzo[1,2-*b*:4,5-*b*']dithiophene-2,6-diyl)bis(trimethylstannane) (0.25 g, 0.26 mmol) and compound 1-3 (0.92 g, 0.64 mmol) were dissolved in 20 mL toluene with degassing by nitrogen for 30 min. After degassing, $Pd_2(dba)_3$ (9.40 mg, 0.010 mmol) and P(*o*-tol)$_3$ (12.50 mg, 0.041 mmol) were added, and the reaction mixture was heated at 110 °C for 12 h. After cooling to room temperature, water was added to the reaction mixture, and the solution was extracted with chloroform before drying over anhydrous $MgSO_4$. After removing the organic solvent, the crude product was purified by column chromatography with chloroform/hexane as an eluent. And the product was purified by recrystallization using chloroform (Yield: 28%). ^{1}H-NMR (1,1,2,2-Tetrachloroethane-D$_2$, 400 MHz): δ (ppm) 7.84 (s, 2H), 7.79-7.77 (d, 4H), 7.65 (s, 2H), 7.38-7.37 (m, 6H), 7.32 (d, 4H), 7.24 (s, 4H), 4.21-4.18 (t, 4H), 2.99-2.90 (m, 20H), 1.91-1.80 (m, 18H), 1.54-1.38 (m, 100H), 1.10-0.97 (m, 54H). MALDI-ToF m/z: 3328.9005. Found: 3329.703 (M^+).

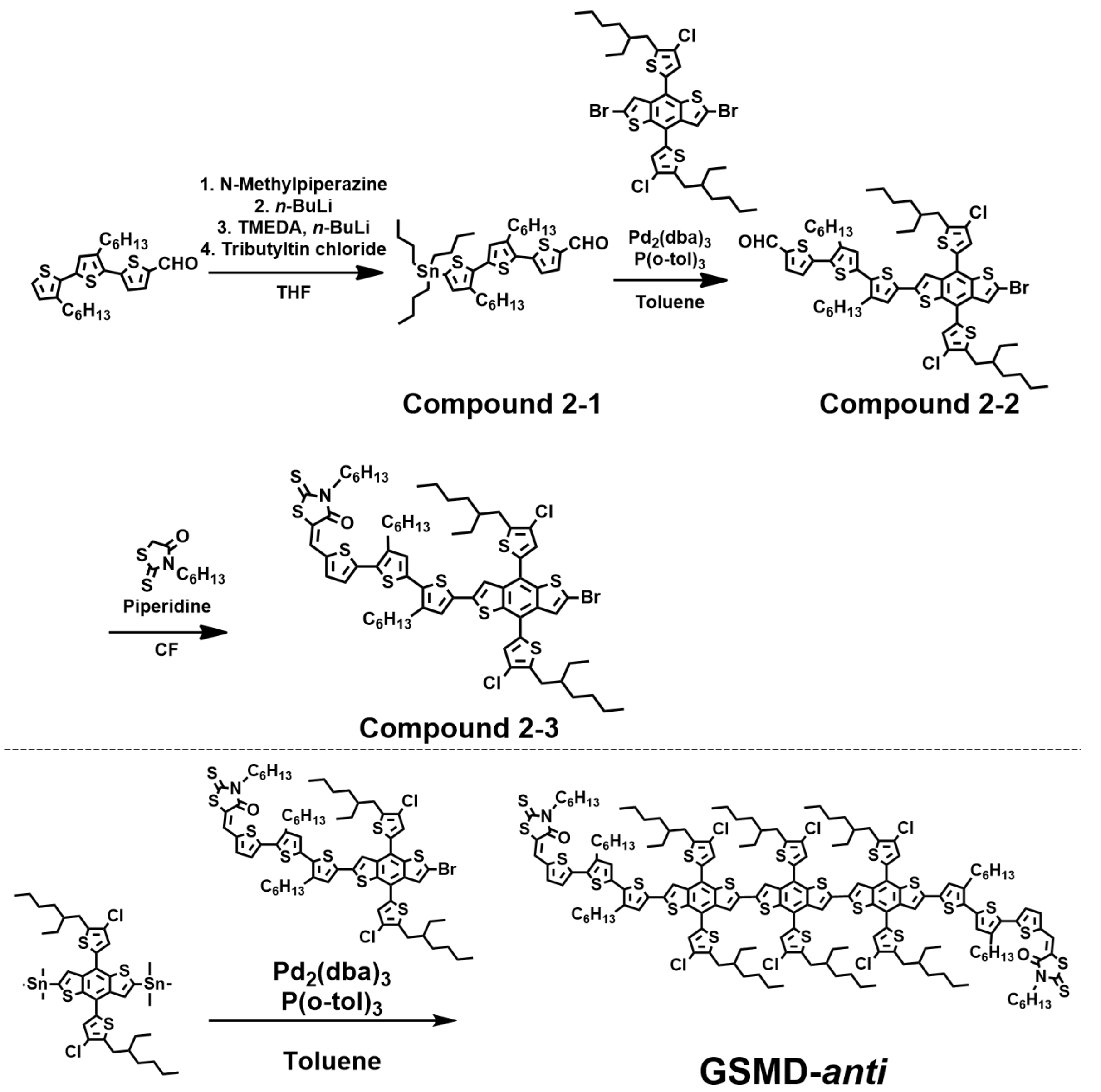

Scheme S2. Synthetic scheme for GSMD-*anti*.

Synthesis of 3',3''-dihexyl-5''-(tributylstannyl)-[2,2':5',2''-terthiophene]-5-carbaldehyde (Compound 2-1)

In a 3-necked flask, *N*-methylpiperazine (1.35 g, 13.50 mmol) was dissolved in THF (160 mL) and cooled to −78 °C. *n*-BuLi (2.50 M, 5.40 mL, 13.50 mmol) was slowly added dropwise, followed by stirring for 30 min. Afterward, 3',3''-dihexyl-[2,2':5',2''-terthiophene]-5-carbaldehyde

(5.00 g, 11.20 mmol) was gradually added dropwise and stirred for an additional 30 min. TMEDA (1.96 g, 16.90 mmol) was then added to the reaction mixture. Subsequently, *n*-BuLi (2.50 M, 6.70 mL, 16.90 mmol) was slowly added dropwise at −78 °C, and the mixture was stirred at a temperature between −70 °C for 2 h. Tributyltin chloride (5.49 g, 16.90 mmol) was added dropwise at −78 °C, and the reaction followed by stirring for 2 h at room temperature. Then, water was added to the reaction mixture, and the solution was extracted with ether before drying over anhydrous $MgSO_4$. Removing the solvent under reduced pressure gave the crude compound 2-1. Without any further purification, the product was used into the next reaction.

Synthesis of 5''-(6-bromo-4,8-bis(4-chloro-5-(2-ethylhexyl)thiophen-2-yl)benzo[1,2-b:4,5-b']dithiophen-2-yl)-3',3''-dihexyl-[2,2':5',2''-terthiophene]-5-carbaldehyde (Compound 2-2)

Compound 2-2 was synthesized by a similar procedure using the compound 2-1 (8.85 g, 2.04 mmol) as compound 1-2 (Yield: 21%). ^{1}H-NMR ($CDCl_3$, 300MHz): δ (ppm) 9.82 (s, 1H) 7.65-7.64 (d, 1H), 7.49-7.47 (d , 2H), 7.19-7.17 (d, 1H), 7.13-7.12 (d, 2H), 7.07 (s, 1H), 6.96(s, 1H), 2.80-2.68 (m, 8H), 1.70-1.57 (m, 6H), 1.41-1.27 (m, 28H) , 0.93-0.80 (m, 18H). ^{13}C-NMR ($CDCl_3$, 400 MHz): 182.60, 146.33, 145.99, 142.59, 142.48, 142.31, 142.13, 141.28, 140.41, 138.88, 138.83, 138.55, 138.42, 137.07, 136.86, 136.60, 136.17, 135.76, 135.09, 134.99, 130.93, 130.28, 129.85, 129.73, 129.27, 129.16, 128.85, 128.10, 125.98, 125.86, 125.78, 124.30, 123.02, 122.95, 122.11, 122.01, 118.38, 117.15, 40.92, 32.54, 32.50, 32.16, 31.66, 30.61, 30.41, 30.25, 29.78, 29.64, 29.37, 29.25, 28.83, 28.30, 27.86, 26.86, 26.79, 25.88, 23.05, 22.61, 17.54, 17.31, 14.18, 14.09, 13.62, 10.92, 10.88.

Synthesis of (E)-5-((5''-(6-bromo-4,8-bis(4-chloro-5-(2-ethylhexyl)thiophen-2-yl)benzo[1,2-b:4,5-b']dithiophen-2-yl)-3',3''-dihexyl-[2,2':5',2''-terthiophen]-5-yl)methylene)-3-hexyl-2-thioxothiazolidin-4-one (Compound 2-3)

Compound 2-3 was synthesized by a similar procedure using compound 2-2 (2.79 g, 1.57 mmol) as compound 1-3 (Yield: 65%). ^{1}H-NMR (CD_2Cl_2, 300MHz): δ (ppm) 7.75 (s, 1H), 7.50-7.49 (d, 2H), 7.32-7.30 (m, 1H), 7.16-7.14 (m, 3H), 7.09 (s, 1H), 6.97 (s, 1H), 4.03-3.98 (t, 2H), 2.81-2.68 (m, 8H), 1.69-1.58 (m, 8H), 1.42-1.18 (m, 34H), 0.93-0.78 (m, 21H). ^{13}C-NMR ($CDCl_3$, 400 MHz): 192.18, 167.54, 144.16, 141.97, 141.16, 140.40, 138.87, 138.82, 138.54, 138.45, 137.22, 137.07, 136.15, 135.40, 135.10, 134.88, 134.66, 131.08, 129.92, 129.28, 128.87, 128.10, 126.66, 125.78, 124.96, 123.02, 122.95, 122.09, 122.00, 120.38, 118.33, 117.12, 44.91, 40.92, 32.54, 32.50, 32.16, 31.70, 31.66, 31.36, 30.38, 30.33, 29.83, 29.67, 29.31, 29.28, 28.83, 26.96, 26.47, 25.90, 23.04, 22.63, 22.52, 14.19, 14.13, 14.02, 10.93, 10.88.

Synthesis of GSMD-anti

(4,8-Bis(4-chloro-5-(2-ethylhexyl)thiophen-2-yl)benzo[1,2-*b*:4,5-*b*']dithiophene-2,6-diyl)bis(trimethylstannane) (0.25 g, 0.26 mmol) and compound 2-3 (0.79 g, 0.58 mmol) were dissolved in toluene (20 mL) with degassing by nitrogen for 30 min. After degassing, $Pd_2(dba)_3$ (9.40 mg, 0.010 mmol) and P(*o*-tol)$_3$ (12.50 mg, 0.041 mmol) were added, and the reaction mixture was heated at 110 °C for 12 h. After cooling to room temperature, water was added to the reaction mixture, and the solution was extracted with chloroform before drying over anhydrous $MgSO_4$. After removing the organic solvent, the crude product was purified by column chromatography with chloroform/hexane as an eluent. And the product was purified by recrystallization using chloroform (Yield: 42%). ^{1}H-NMR (1,1,2,2-Tetrachloroethane-D_2, 400 MHz): δ (ppm) 7.89(s, 2H),

7.76-7.74 (d, 4H), 7.62 (s, 2H), 7.43-7.42 (d, 2H), 7.37-7.36 (m, 6H), 7.30-7.29 (d, 2H), 7.20 (s, 2H), 7.10 (s, 2H), 4.21-4.18 (t, 4H), 3.00-2.85 (m, 20H), 1.91-1.77 (m, 18H), 1.46-1.44 (m, 84H), 1.10-1.00 (m, 54H). MALDI-ToF m/z: 3216.7753. Found: 3216.792 (M^+).

Characterizations: Bruker AVANCE NEO (9.4 T) spectrometer was used to obtain the nuclear magnetic resonance (NMR) spectra of the materials, and the chemical shifts in the spectra have units of ppm. Matrix-assisted laser desorption/ionization time of flight (MALDI-ToF) spectra was measured by Autoflex maX from Bruker. The optimized molecular structures and the relaxed potential surface energy from bond rotation were computed using the density functional theory (DFT) method with the Becke three-parameter Lee-Yang-Parr (B3LYP) function and the 6-31G* basis set *via* a modeling software (Gaussian 09).

Cyclic voltammetry (CV) was performed using a EG and G Parc model 273 Å potentiostat/galvanostat system in a 0.1 M tetrabutylammonium perchlorate solution with nitrogen degassed anhydrous acetonitrile as the supporting electrolyte, at a scan rate of 50 mV s^{-1}. A glassy carbon electrode was used as the working electrode. A platinum wire was used as the counter electrode, and an Ag/AgCl electrode was used as the reference electrode. The redox couple ferricenium/ferrocene was used as external standard. Frontier orbital energy levels of the materials were estimated from cyclic voltammetry: E_{HOMO} (eV) = $-(E_{\mathrm{onset}}^{\mathrm{ox.}} - E_{\mathrm{onset}}^{\mathrm{Fc/Fc+}}) + E_{\mathrm{HOMO}}^{\mathrm{Fc}}$; E_{LUMO} (eV) = $-(E_{\mathrm{onset}}^{\mathrm{red.}} - E_{\mathrm{onset}}^{\mathrm{Fc/Fc+}}) + E_{\mathrm{HOMO}}^{\mathrm{Fc}}$; $E_{\mathrm{onset}}^{\mathrm{Fc/Fc+}} = 0.44$ eV, $E_{\mathrm{HOMO}}^{\mathrm{Fc}} = -4.8$ eV.

A UV-1800 spectrophotometer was used for the ultraviolet-visible (UV–vis) absorption spectra. The differential scanning calorimetry (DSC) profiles were recorded by TA Instruments DSC 25 with heating and cooling rates of 10 °C min^{-1} from 20 to 400 °C for pristine and blend films. Thermo gravimetric analysis (TGA) was performed using TGA N-1000 (SCINCO Co.). The

atomic force microscopy (AFM) images were measured by MultiMode 8-HR from Bruker to measure the morphology and thickness of films. K3100 IQX, McScience Inc. Instrument, was used to analyze the EQE spectra, equipped with a monochromator (Newport) and an optical chopper (MC 2000 Thorlabs).

The resonant soft X-ray scattering (RSoXS) experiment was performed at beamline 11.0.1.2 in the S11 Advanced Light Source (United States). Blend films for the RSoXS measurement were prepared on a 100 nm-thick, 1.0 mm × 1.0 mm Si_3N_4 membrane supported by a 200-μm thick, 5 mm × 5 mm silicon frame (Norcada Inc.). The domain size of a blend film was approximated to be half of the domain spacing (domain spacing = $2\pi q_{\mathrm{peak}}{}^{-1}$) from the RSoXS profile. The relative domain purity was estimated as the relative ratio of square-root of the integrated scattering intensity in the Iq^2 vs. q plot. Grazing incidence X-ray scattering (GIXS) measurements were conducted at the Pohang Accelerator Laboratory (beamline 9A, Republic of Korea), with incidence angles between 0.12 – 0.14°. Correlation length (L_c) values of the crystallites were calculated using the Scherrer equation:

$$L_{\mathrm{c}} = \frac{2\pi K}{\Delta q}$$

(K (shape factor) = 0.9 and Δq = full width half maximum (FWHM) of the scatterings)

Space-charge limited current (SCLC) mobility measurements: The electron mobilities (μ_es) for blend films and the hole mobilities (μ_hs) of the neat donor films and blend films were estimated from the SCLC method. The hole-only devices with a structure of indium tin oxide (ITO)/poly(3,4-ethylenedioxythiophene):poly(styrenesulfonate) (PEDOT:PSS)/pristine donors or active layer/Au were fabricated to estimate μ_h values. For estimation of μ_e values, the electron-only devices with a

structure of ITO/ZnO/active layer/poly[9,9-bis(3′-(*N*,*N*-dimethyl)-*N*-ethylammoinium-propyl-2,7-fluorene)-*alt*-2,7-(9,9-dioctylfluorene)]dibromide (PFN-Br)/Al were fabricated. The preparation for active layers followed the same conditions as those described for the OSC fabrications. The current-voltage measurements were conducted with the applied voltage range of 0 to 6 V, and the obtained results were fitted using the Mott-Gurney law.[3]

$$J = \frac{9\varepsilon_r\varepsilon_0\mu V^2}{8L^3}$$

where J denotes the current density, ε_r represents the relative dielectric constant of the films, ε_0 exhibits the permittivity of free space (8.85×10^{-14} F cm^{-1}), μ is the charge carrier (hole or electron) mobility, V is the calculated potential across the SCLC device ($V = V_{applied} - V_{bi} - V_r$, where V_{bi} is the built-in state potential and V_r is the voltage drop resulting from resistance), and L is the thickness of the blend or pristine films measured by AFM.

Estimation of glass transition temperature: Glass transition temperature (T_g) was evaluated using UV−vis spectroscopy. Absorption spectra of BTR-Cl and GSMDs films were measured while heating from 20 to 190 °C. To ensure consistency with the OSC devices, donor films were prepared under identical conditions regarding solvent, concentration, and spin-coating parameters. The deviation metric (DM_T) of each absorption spectra was calculated, follwing the approach described by Samuel E. Root *et al.*:[4]

$$\boldsymbol{DM_T = \sum_{\lambda_{min}}^{\lambda_{max}} [I_{RT}(\lambda) - I_T(\lambda)]^2}$$

where λ represents the wavelength within the optical sweep range defined by λ_{max} and λ_{min}. $I_{RT}(\lambda)$ and $I_T(\lambda)$ denote the normalized absorption intensities of the film at room temperature and at temperature T, respectively. Then, the T_g is assigned as the temperature corresponding to the

intersection point of two linear fits applied to the low- and high-temperature regimes of the DM_T curve.

Organic solar cell (OSC) fabrication and characterization: The OSCs with a conventional architecture of ITO/PEDOT:PSS (or Br-2PACz)/active layer/PNDIT-F3N-Br/Ag were prepared with the following procedures. ITO-coated glass substrates were treated by ultrasonication with acetone, deionized water, and isopropyl alcohol. Then, the ITO substrates were dried for 6 h in an oven (70 °C) at an ambient pressure. For PEDOT:PSS-based devices, the ITO substrates were plasma treated for 10 min and then spin-coating of the PEDOT:PSS solution (Clevios, AI4083) was performed at 3300 rpm for 30 s onto the ITO substrates. Then, the film/substrate was annealed in the air at 160 °C for 15 min before transferring into an N_2−filled glovebox. For Br-2PACz-based devices, the ITO substrates were plasma treated for 1 min and then Br-2PACz solution (0.5 mg mL^{-1} in 1-butanol:ethanol (5:1 v/v) solution) was spin-coated onto ITO substrates under 7000 rpm for 60 s in N_2−filled glovebox and annealed at 110 °C for 10 min. The active layer solution for the BTR-Cl:Y6 blend system consisting the 1.6:1 weight ratio of donor to acceptor was prepared as 16.0 mg mL^{-1} concentration in chloroform (CF) without additive spin-coated onto the PEDOT:PSS/ITO substrate at 50 °C to form an active layer with a thickness of ~110 nm. In similar way, the active layer solutions for the GSMD-*syn* and GSMD-*anti* were prepared using donor-to-acceptor weight ratios of 2:1 and 1.5:1, respectively. Then, the samples were thermally annealed at 120 °C for 10 min and dried with high vacuum ($<10^{-6}$ Torr) for 1 h. PNDIT-F3N-Br in methanol (1 mg mL^{-1}) was then spin-coated with the condition of 3000 rpm for 30 s. Finally, Ag (120 nm) was deposited under high vacuum ($<10^{-6}$ Torr) in an evaporation chamber. Optical microscopy (OM) was used to measure the exact photoactive area of the mask (0.044 cm^2). Keithley 2400

SMU instrument was used to measure the PCE values under an Air Mass 1.5 G solar simulator (100 mW cm^{-2}, solar simulator: K201 LAB55, McScience). K801SK302 from McScience, satisfying the Class AAA, ASTM Standards, was used as a standard silicon reference cell to calibrate the exact solar intensity.

Photostability test of the OSCs: Photostability of the OSCs was measured under 1-sun illumination (100 mW cm^{-2}) within an N_2-filled glove box. A Xenon lamp (ABET Technology Inc.) was used for irradiation without blocking ultraviolet light. A silicon solar cell (K801SK302) from McScience served as the standard cell for calibrating the precise solar intensity. The OSCs featuring a normal type (ITO/PEDOT:PSS/active layer/PNDIT-F3N-Br/Ag) without encapsulation were illuminated for 1000 h to conduct the photostability test at an ambient temeprature. Subsequent measurements were examined using the same instruments and methods as described above. Data for each point represent the average from more than three independent devices.

Supporting Figures & Tables

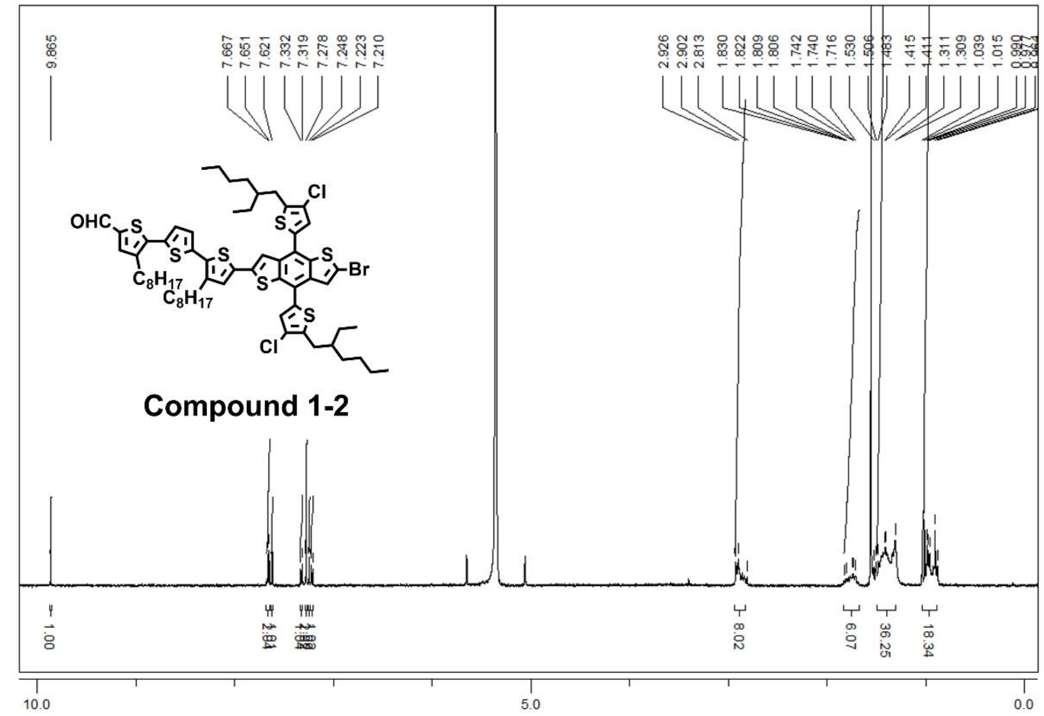

Figure S1. ^{1}H NMR spectrum of compound 1-2.

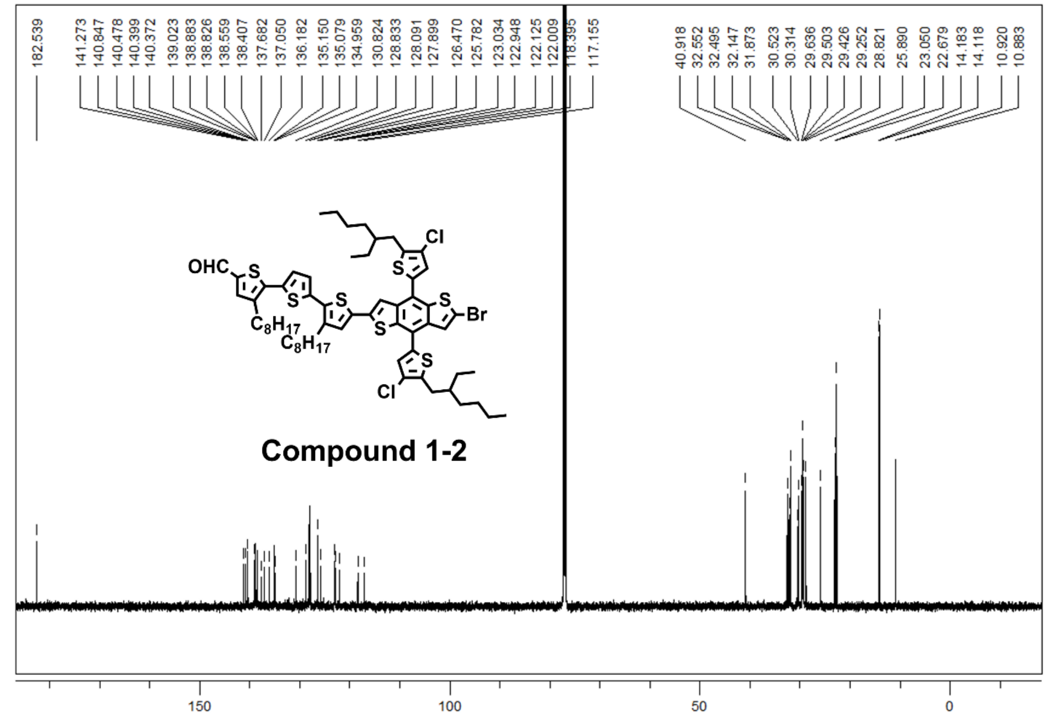

Figure S2. ^{13}C NMR spectrum of compound 1-2.

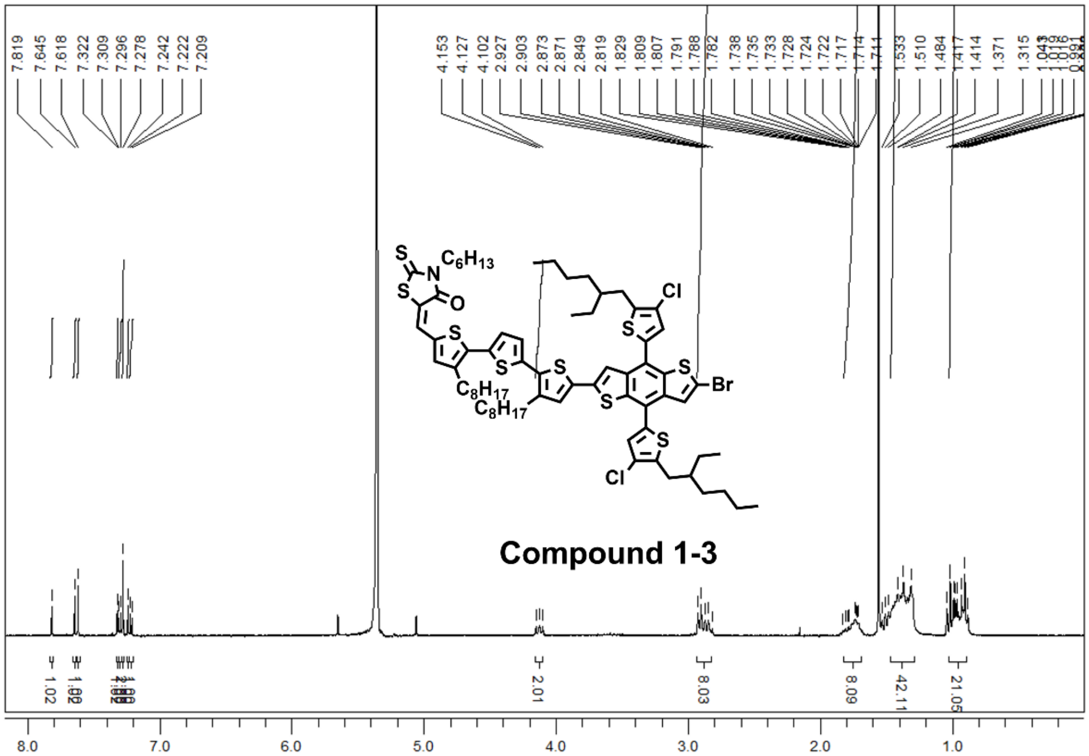

Figure S3. ^{1}H NMR spectrum of compound 1-3.

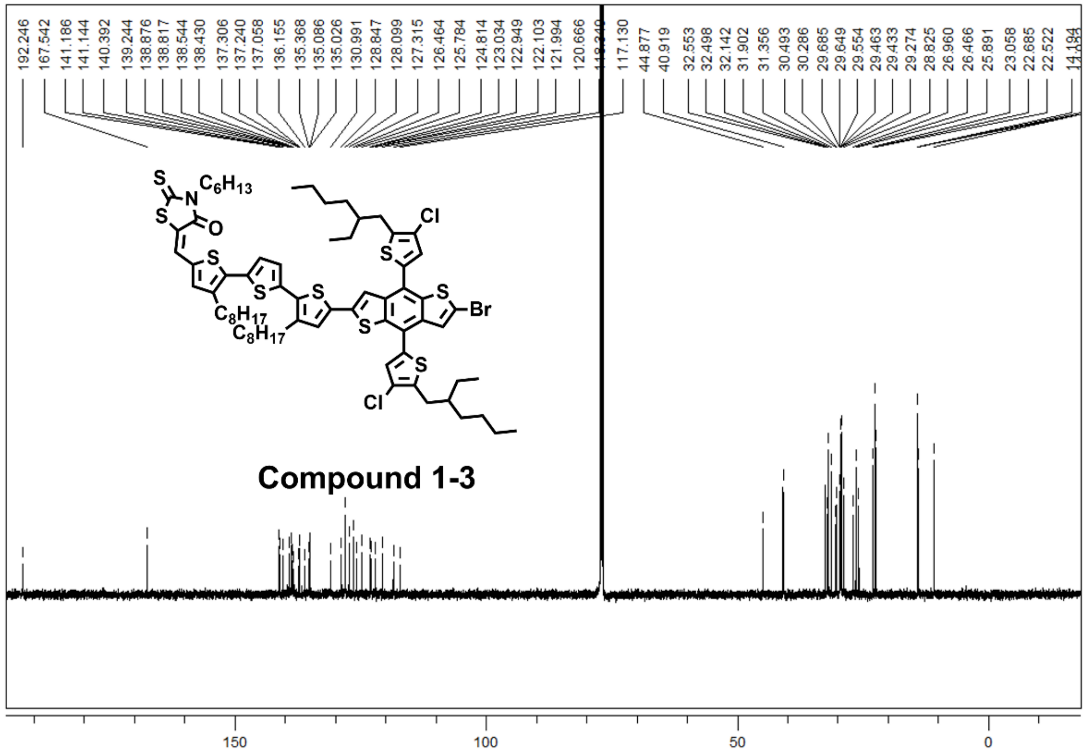

Figure S4. ^{13}C NMR spectrum of compound 1-3.

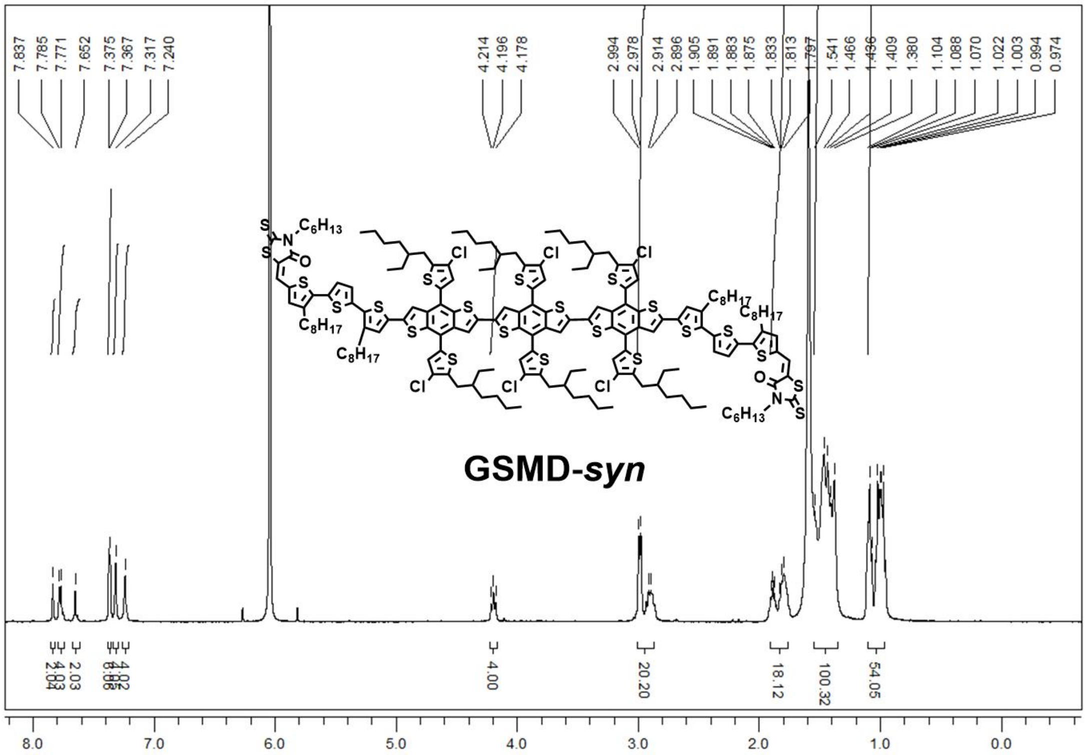

Figure S5. ^{1}H NMR spectrum of GSMD-*syn*.

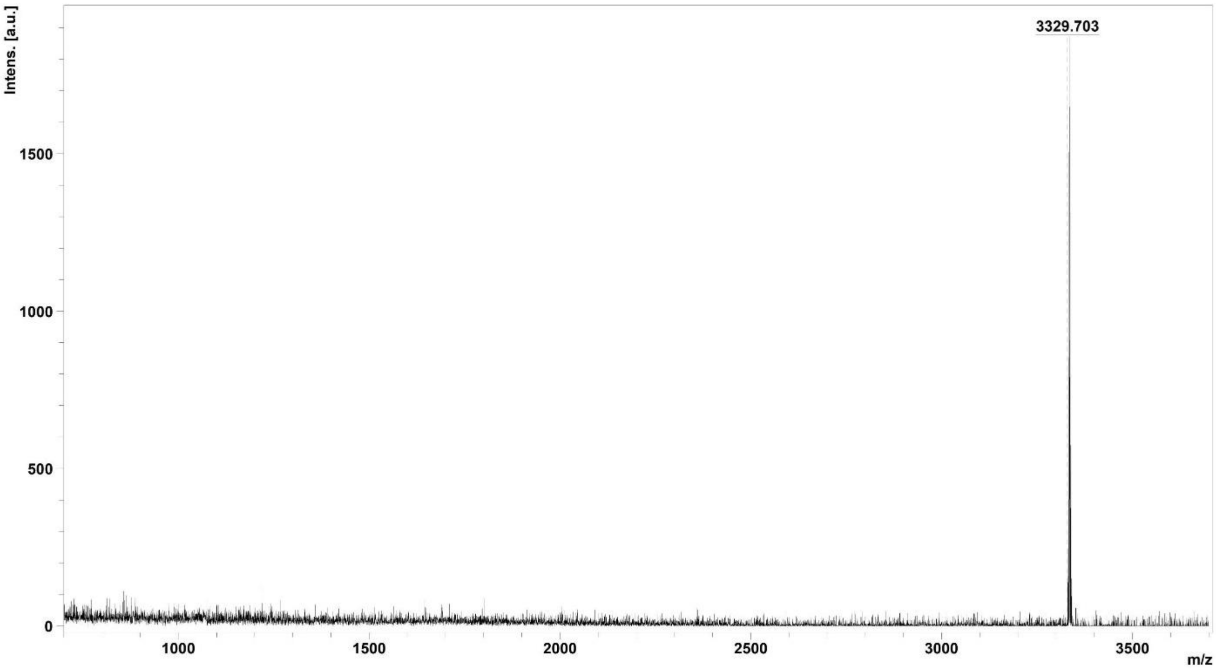

Figure S6. MALDI-ToF spectrum of GSMD-*syn*.

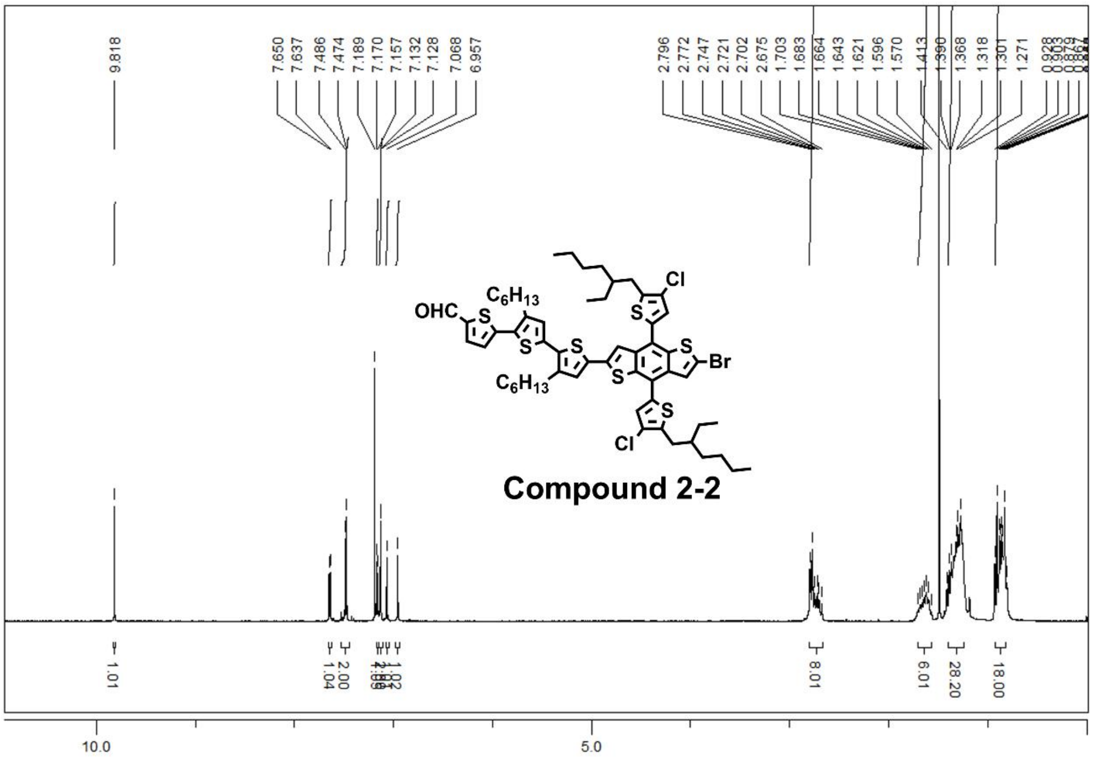

Figure S7. ^{1}H NMR spectrum of compound 2-2.

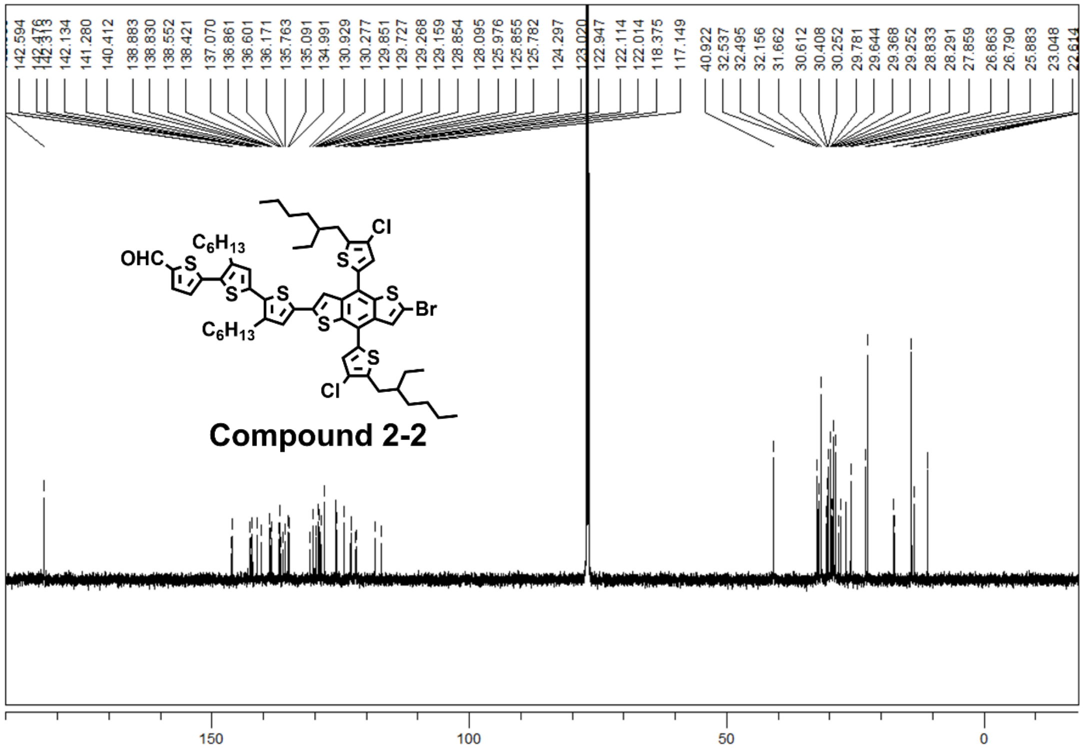

Figure S8. ^{13}C NMR spectrum of compound 2-2.

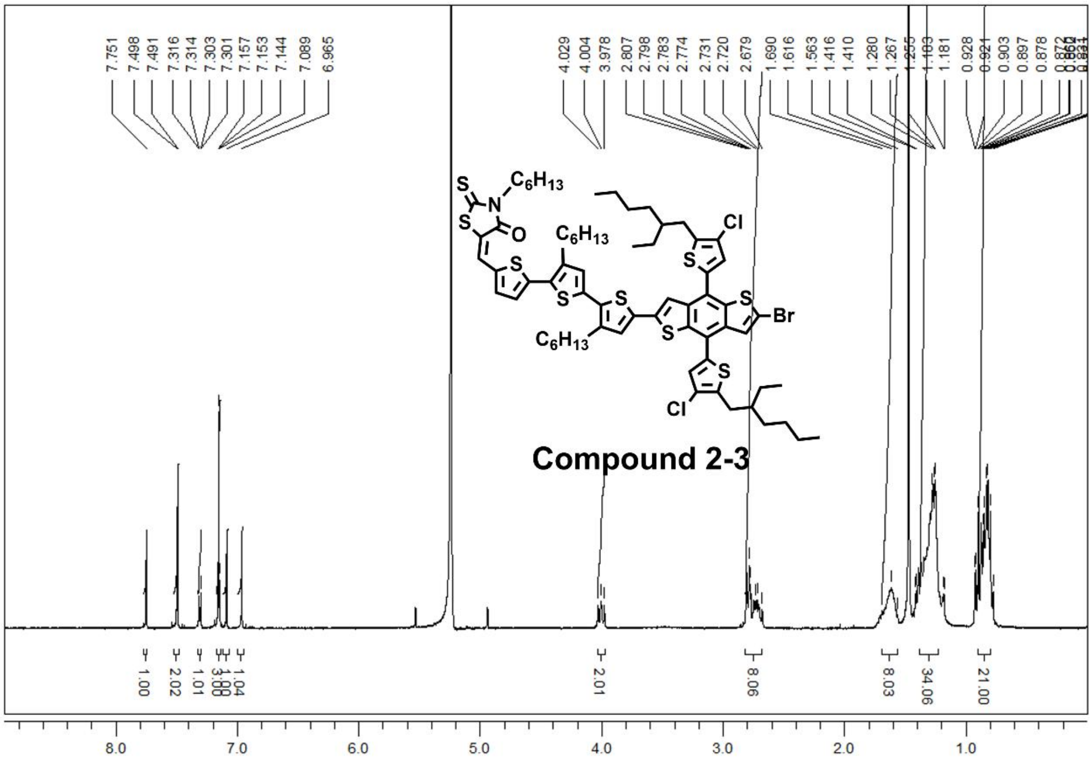

Figure S9. ^{1}H NMR spectrum of compound 2-3.

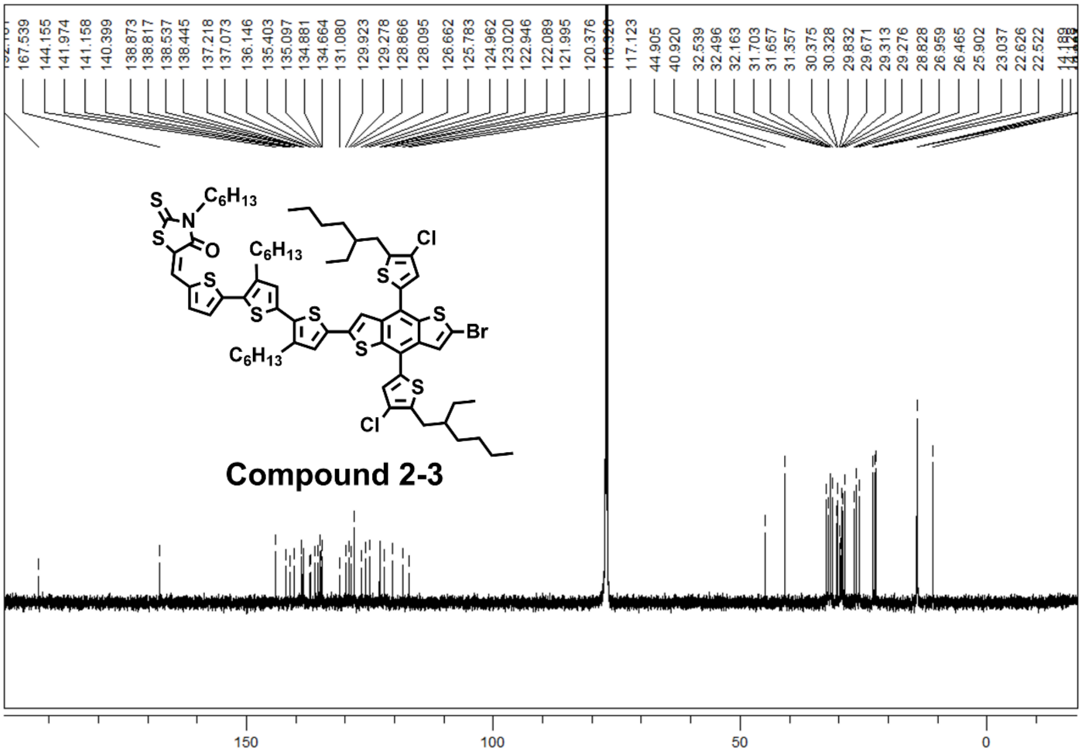

Figure S10. ^{13}C NMR spectrum of compound 2-3.

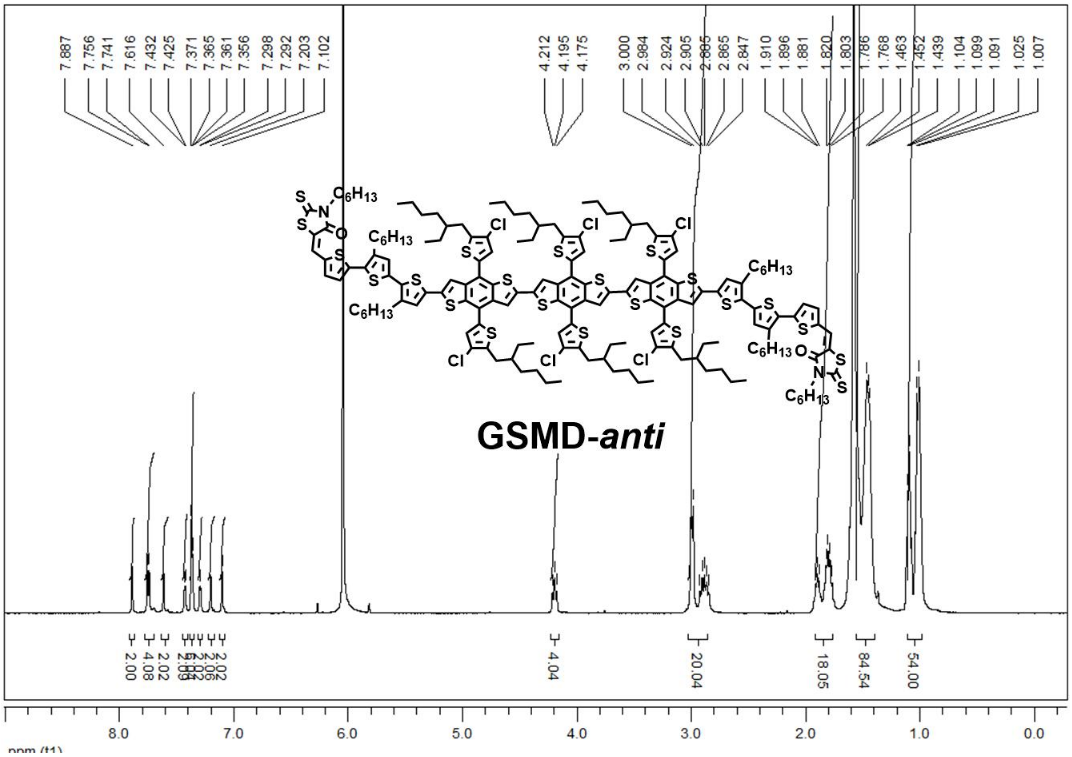

Figure S11. ^{1}H NMR spectrum of GSMD-*anti*.

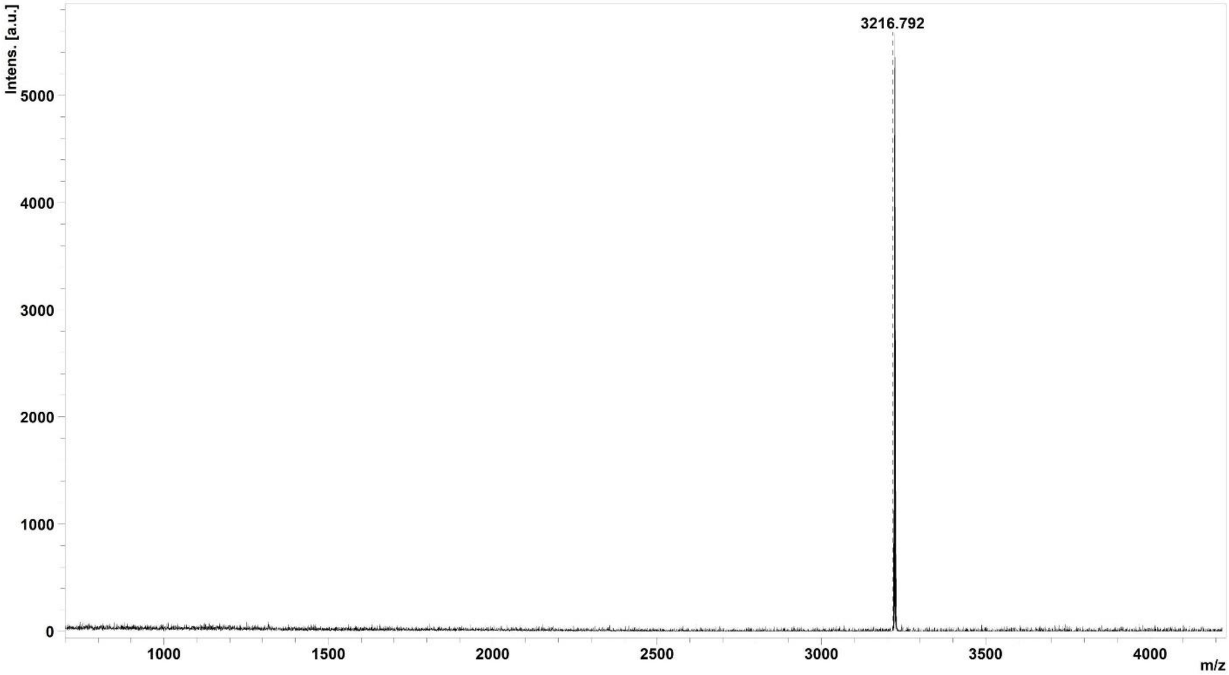

Figure S12. MALDI-ToF spectrum of GSMD-*anti*.

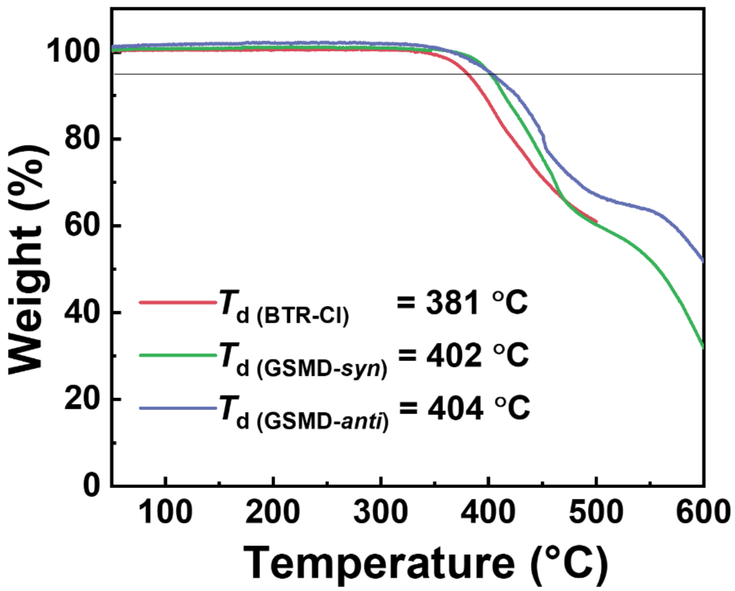

Figure S13. TGA spectra of BTR-Cl and GSMDs.

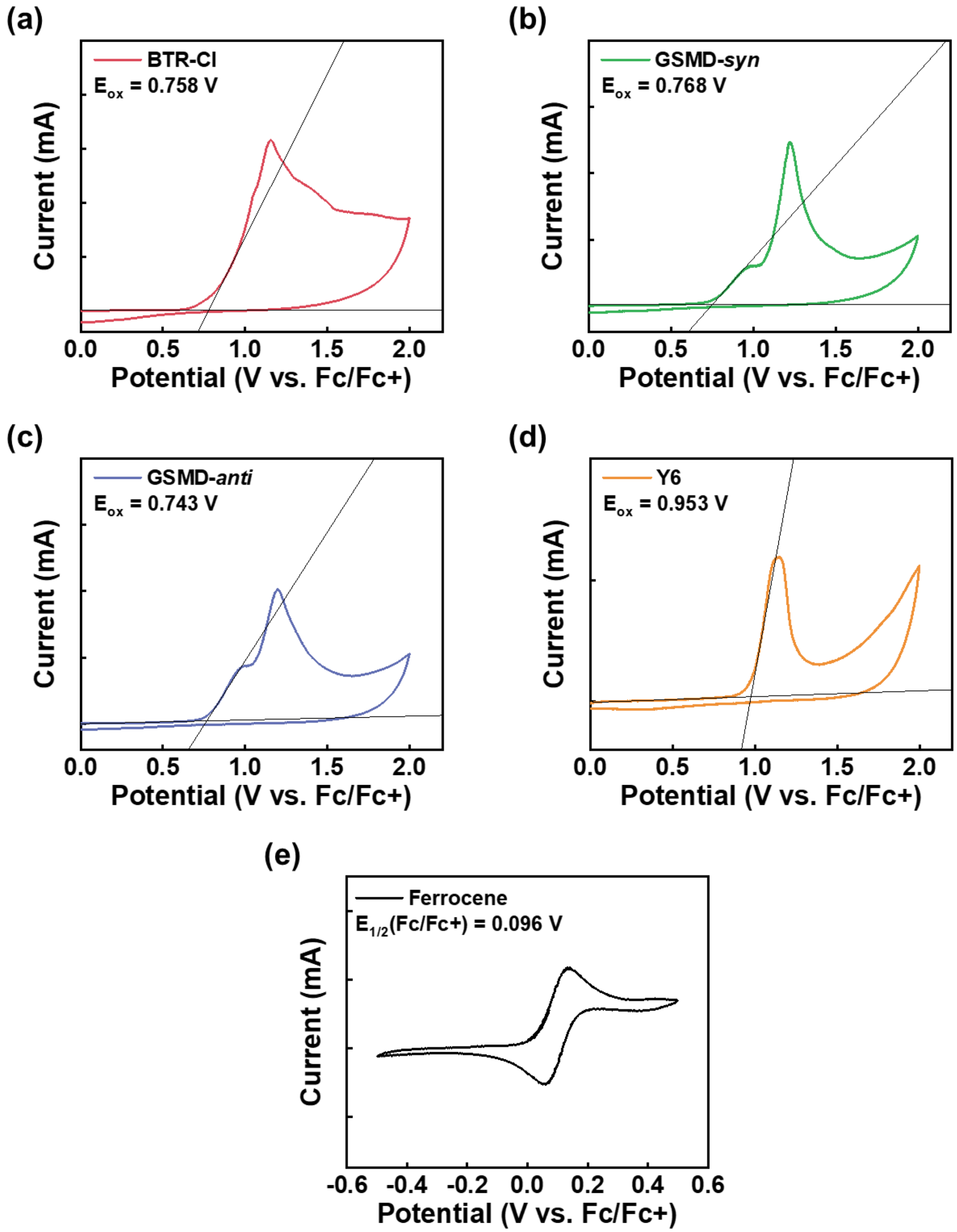

Figure S14. CVs of (a) BTR-Cl, (b) GSMD-*syn*, (c) GSMD-*anti*, (d) Y6, and (e) ferrocene.

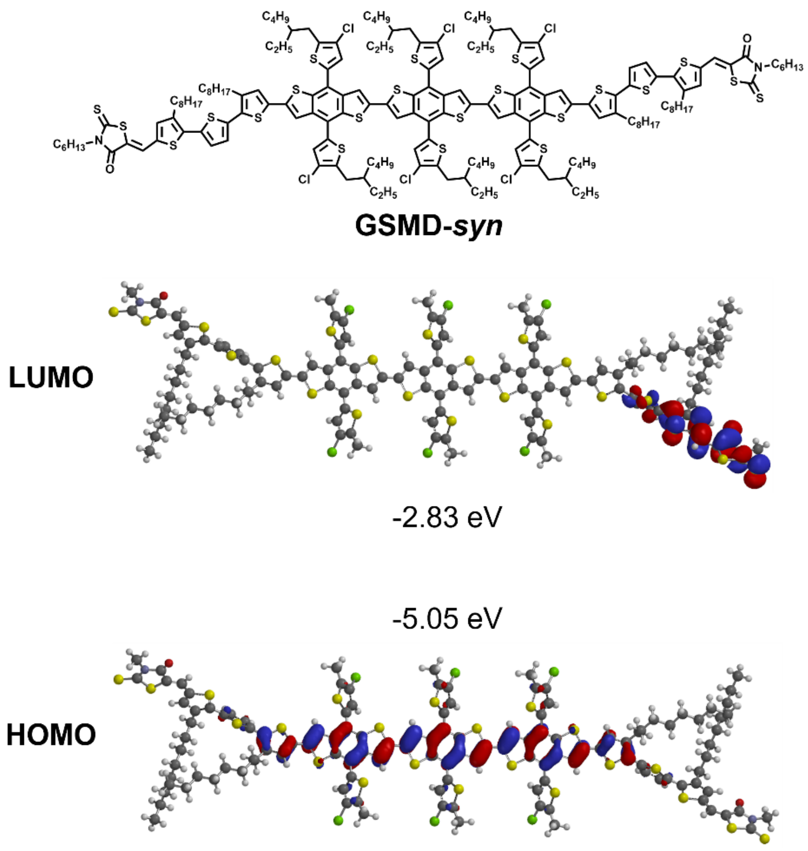

Figure S15. The frontier molecular orbitals (HOMO and LUMO) from GSMD-*syn* calculated based on DFT simulations.

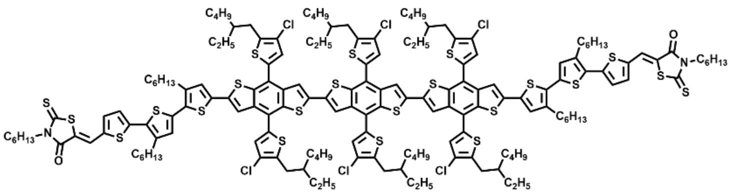

GSMD-*anti*

LUMO

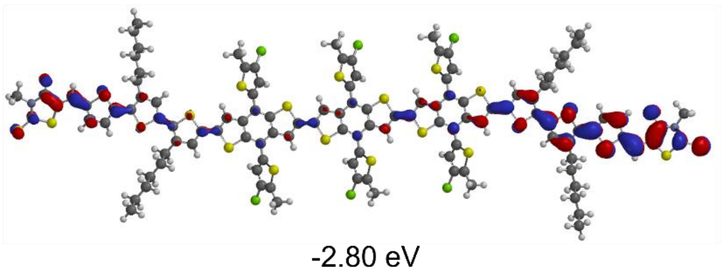

-2.80 eV

-5.03 eV

HOMO

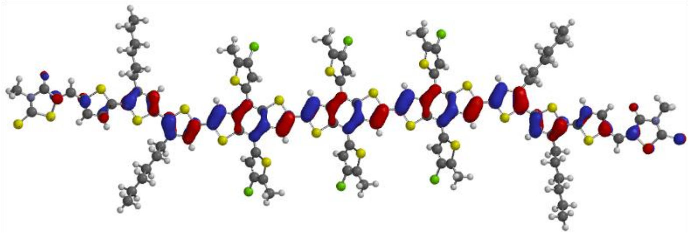

Figure S16. The frontier molecular orbitals (HOMO and LUMO) from GSMD-*anti* calculated based on DFT simulations.

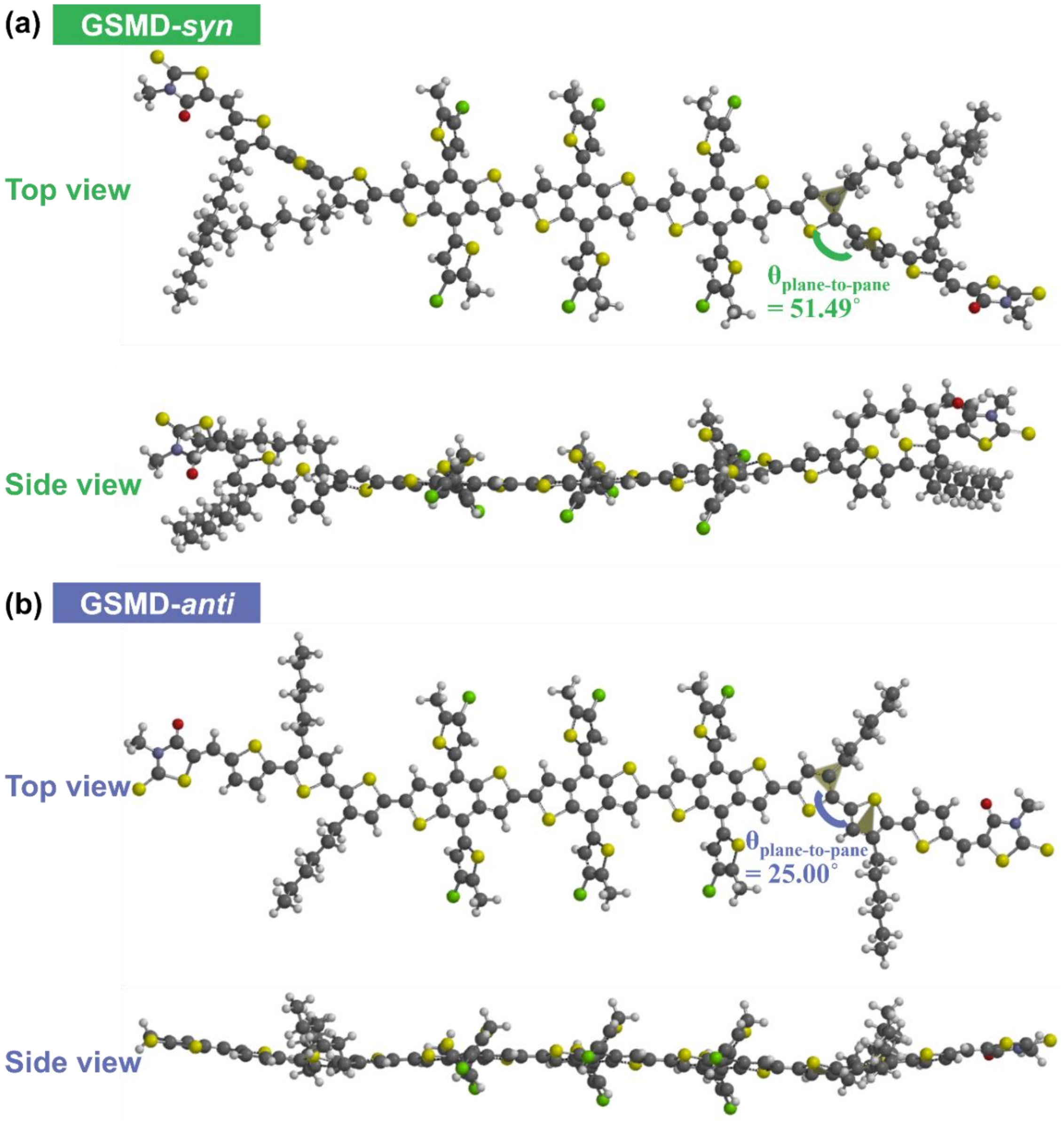

Figure S17. Optimized molecular conformations of GSMDs; (a) GSMD-*syn* and (b) GSMD-*anti* obtained from DFT simulations.

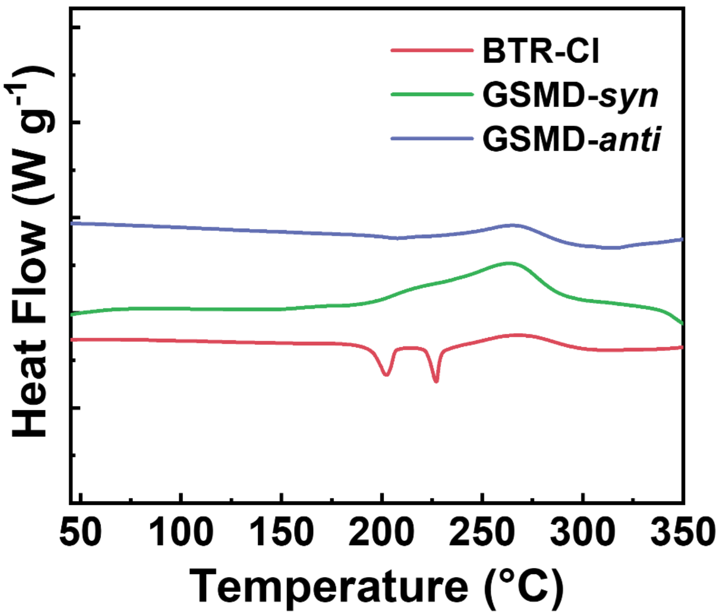

Figure S18. DSC thermograms of pristine donor films obtained during the 1st heating cycle at a rate of 10 °C min^{-1}.

Table S1. DSC results and SCLC hole mobilities of the pristine donor films.

Donor	T_m [°C]	ΔH_m [J g^{-1}]	T_{cc} [°C]	ΔH_{cc} [J g^{-1}]	μ_h[a] [cm^2 V^{-1} s^{-1}]
BTR-Cl	202.3, 227.1	14.6, 10.3	-	-	2.9×10^{-4}
GSMD-*syn*	-	-	264.0	100.1	1.6×10^{-4}
GSMD-*anti*	-	-	265.7	65.5	2.0×10^{-4}

[a]Average values obtained from at least 3 independent devices.

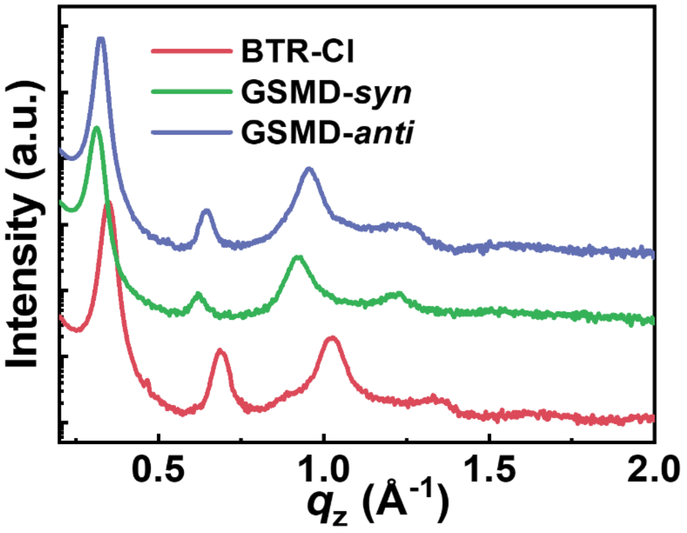

Figure S19. GIXS line-cut profiles in the out-of-plane direction of the pristine donors.

Table S2. SCLC mobilities for the donors:Y6 blend films.

Donor	μ_e[a] [cm^2 V^{-1} s^{-1}]	μ_h[a] [cm^2 V^{-1} s^{-1}]	μ_e/μ_h
BTR-Cl	2.8 × 10^{-4}	2.1 × 10^{-4}	1.3
GSMD-*syn*	2.4 × 10^{-4}	1.0 × 10^{-4}	2.3
GSMD-*anti*	2.7 × 10^{-4}	2.6 × 10^{-4}	1.1

[a]Average values obtained from at least 3 independent devices.

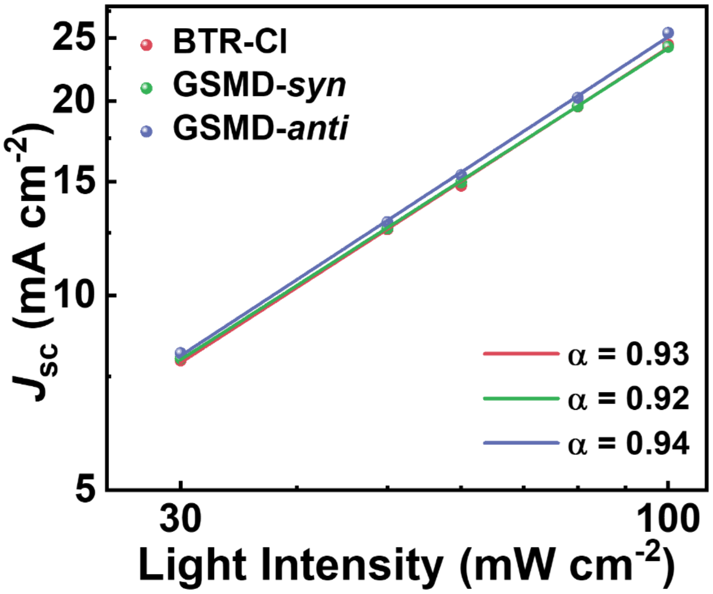

Figure S20. Light intensity-dependent J_{sc} plots of Y6-based OSCs.

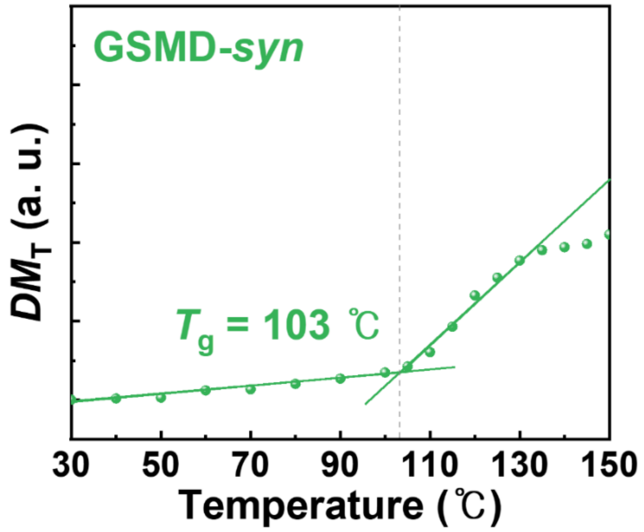

Figure S21. DM_T plot of GSMD-*syn* films as a function of temperature.

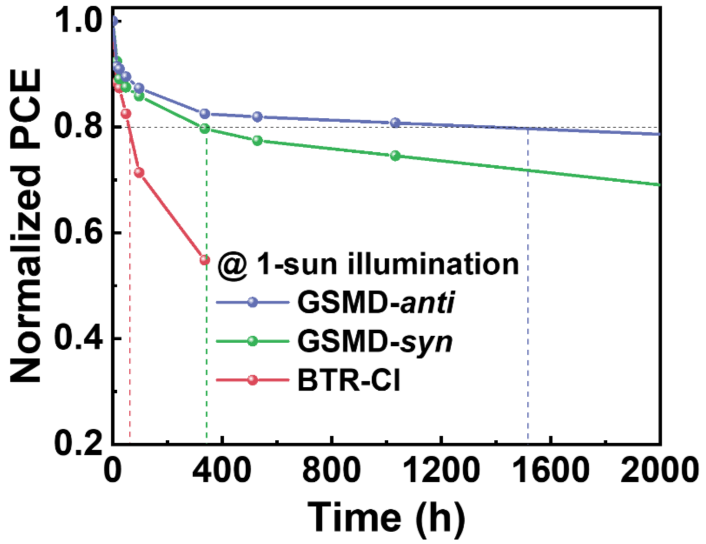

Figure S22. Normalized PCE values of binary blend system under 1-sun illumination.

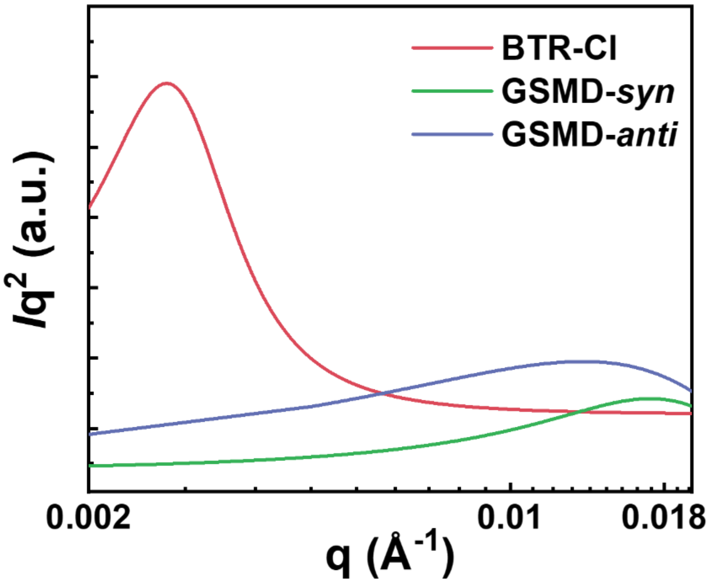

Figure S23. RSoXS profiles of blend films.

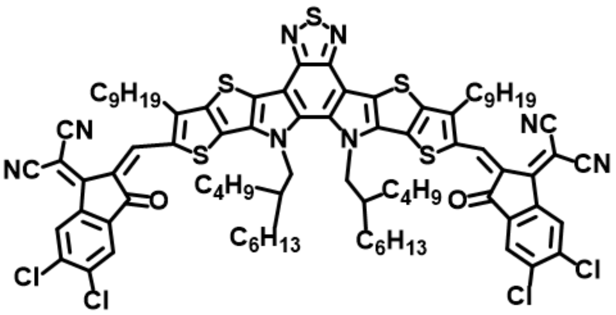

Figure S24. Chemical structures of BTP-eC9.

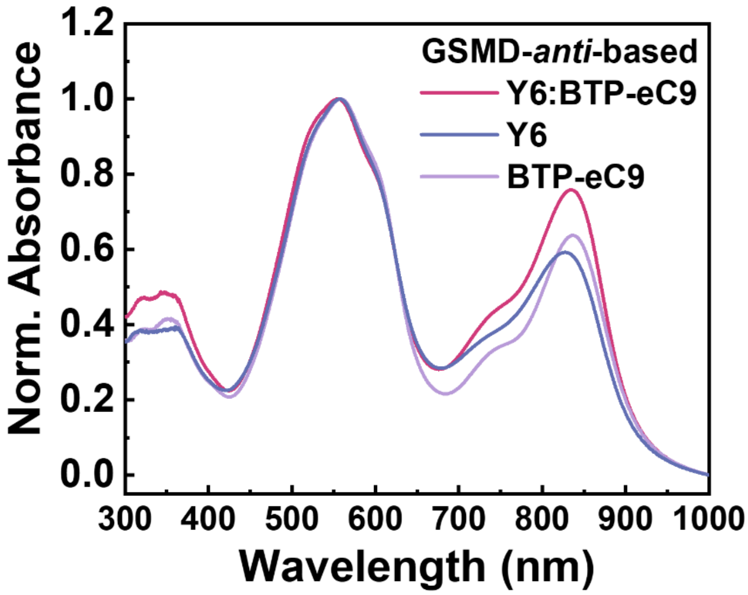

Figure S25. UV−vis absorption spectra in film of GSMD-*anti*-based blends.

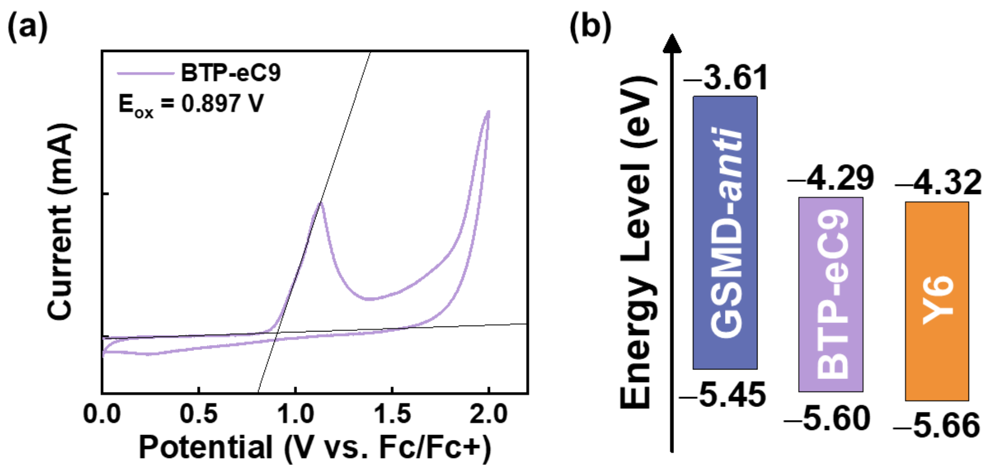

Figure S26. (a) CV of BTP-eC9 and (b) energy level alignment in film of donor and acceptors.

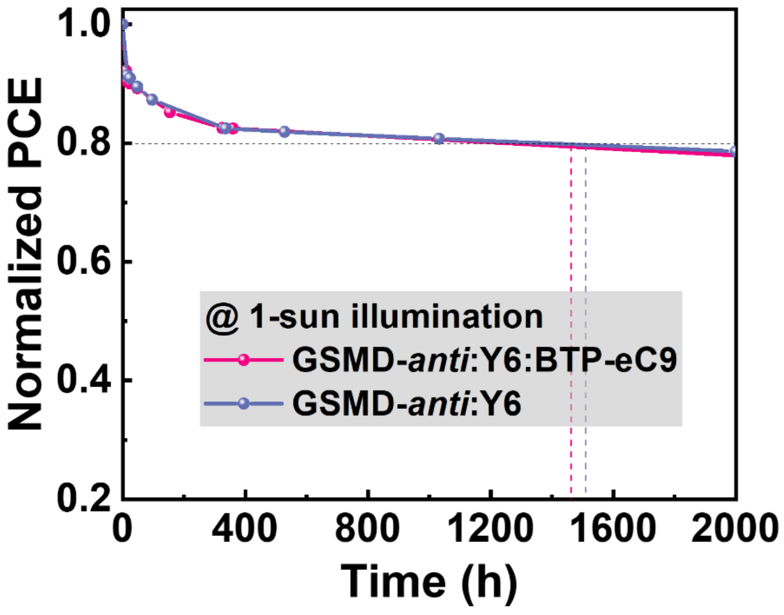

Figure S27. Normalized PCE values under 1-sun illumination.

References

[1] a) R. J. Kumar, J. M. MacDonald, T. B. Singh, L. J. Waddington, A. B. Holmes, *J. Am. Chem. Soc.* **2011**, *133*, 8564; b) S. Zhang, Y. Qin, J. Zhu, J. Hou, *Adv. Mater.* **2018**, *30*, 1800868; c) S. Seo, J.-Y. Park, J. S. Park, S. Lee, D.-Y. Choi, Y.-H. Kim, B. J. Kim, *Nano Res. Energy* **2024**, *3*, e9120088; d) L. Yang, S. Zhang, C. He, J. Zhang, Y. Yang, J. Zhu, Y. Cui, W. Zhao, H. Zhang, Y. Zhang, Z. Wei, J. Hou, *Chem. Mater.* **2018**, *30*, 2129; e) J. Hong, J. Y. Choi, T. K. An, M. J. Sung, Y. Kim, Y.-H. Kim, S.-K. Kwon, C. E. Park, *Dyes Pigm.* **2017**, *142*, 516.

[2] Z. Wu, C. Sun, S. Dong, X.-F. Jiang, S. Wu, H. Wu, H.-L. Yip, F. Huang, Y. Cao, *J. Am. Chem. Soc.* **2016**, *138*, 2004.

[3] Z. Chiguvare, V. Dyakonov, *Phys. Rev. B* **2004**, *70*, 235207.

[4] S. E. Root, M. A. Alkhadra, D. Rodriquez, A. D. Printz, D. J. Lipomi, *Chem. Mater.* **2017**, *29*, 2646.
